# Non-spatial and spatial heterogeneity revealed a suppressive immune feature of Siglec-15 in lung adenocarcinomas

**DOI:** 10.1186/s12967-023-04489-6

**Published:** 2023-09-06

**Authors:** Baihui Li, Yan Guo, Yeran Yi, Ziqi Huang, Yulin Ren, Hao Wang, Lili Yang

**Affiliations:** 1 Department of Immunology, Tianjin Medical University Cancer Institute and Hospital, Huanhuxi Road, Tiyuanbei, Hexi District, Tianjin, 300060 People’s Republic of China; 2 Tianjin Medical University Cancer Institute & Hospital, National Clinical Research Center for Cancer, Tianjin, China; 3 Key Laboratory of Cancer Immunology and Biotherapy, Tianjin, China; 4 Tianjin’s Clinical Research Center for Cancer, Tianjin, China; 5 Department of Esophageal Cancer, Tianjin Medical University Cancer Institute and Hospital, Tianjin, China

**Keywords:** Siglec-15, CD8^+^ T cells, Spatial heterogeneity, Immune suppression, Immunotyping, Lung adenocarcinoma

## Abstract

**Background:**

Sialic acid-binding immunoglobulin-like lectin-15 (Siglec-15) has emerged as a novel immunotherapy candidate, which deserves a comprehensive investigation in lung adenocarcinoma (LUAD).

**Methods:**

Multiplex fluorescence‐based immunohistochemistry was conducted to assess Siglec-15 expression and tumor-infiltrating immune cells in LUAD from Tianjin cohort, with validation cohorts Xinchao 04 and 07.

**Results:**

This study revealed that Siglec-15 was positively correlated with CD8^+^ T cells and tumor-associated macrophages (TAMs) infiltration, but CD8^+^ T cells were mostly infiltrated in the stroma area, not in the tumor area. Spatially, fewer CD8^+^ T cells surrounded Siglec-15^+^ tumor cells in PD-L1^−^ cells, and more TAMs surrounded Siglec-15^+^ tumor cells in PD-L1^−/+^ cells. Siglec-15^+^ TAMs infiltrated with more CD8^+^ T cells, and were closer to CD8^+^ T cells than Siglec-15^−^ TAMs and Siglec-15^+^ tumor cells. Siglec-15^+^ TAMs infiltrated with more Tregs and were closer to Tregs than Siglec-15^+^ tumor cells. Siglec-15^+^ tumor cells or TAMs reversed CD8^+^ T cells prognosis value, and enhanced the prognosis value of Tregs and TAMs. The immunotyping based on Siglec-15 and CD8A / CD8^+^ T cells revealed that patients with high CD8A and Siglec-15 expression exhibited immune activation. Patients with low CD8A expression / CD8^+^ T cells infiltration and Siglec-15 overexpression were related to the activation of immunosuppressive signature and metabolism-related pathway, and infiltrated with more TAMs.

**Conclusions:**

We revealed the distinct characteristics between Siglec-15^+^ tumor cells and TAMs in relation to CD8^+^ T cells, and a unique relationship between Siglec-15 and immunosuppressive TIME in LUAD, which may provide potential value for anti-Siglec-15 therapy.

**Supplementary Information:**

The online version contains supplementary material available at 10.1186/s12967-023-04489-6.

## Background

In 2011, ipilimumab was approved as an immunotherapy for malignancies, introducing a new chapter in cancer treatment [1]. Compared to traditional cancer therapies, immunotherapy was generally better tolerated and has higher objective remission rates, improving patients’ overall survival (OS) [2]. Recently, immune checkpoint inhibitors (ICIs), such as programmed death-1 receptor (PD-1)/programmed death ligand-1 (PD-L1), have been implicated in many solid tumors, including malignant melanoma and non-small cell lung cancer (NSCLC), and have achieved good therapeutic effects [3, 4]. ICIs have rapidly evolved from clinical trial drugs to first- and second-line therapeutic agents. ICIs monotherapies or combination therapies have completely revolutionized NSCLC treatment [5], gradually realizing the dream of a clinical cure for patients with advanced disease [6], and have also improved the treatment status of patients with early-stage lung cancer [7]. However, the current overall efficacy of anti-PD-1/PD-L1 immunotherapies in solid tumors is less than 30% [6, 8], indicating that the PD-1/PD-L1 pathway is not the only one mechanism by which tumor immune escape occurs. Hence, other possible immunosuppressive pathways exist in the complex tumor immune microenvironment (TIME) network [9]. Therefore, it is imperative to identify novel immune checkpoints as supplements to anti-PD-1/PD-L1 immunotherapy.

Sialic acid-binding immunoglobulin-like lectins (Siglecs), which specifically recognize sialic acid structures, play an important regulatory role in innate and adaptive immunity [10]. In recent years, an increasing number of Siglecs family members have been identified to play crucial roles in tumor immunosuppression [11, 12]. Siglec-15 (gene name: *SIGLEC15*) is originally defined as a member of the Siglecs family and mainly regulates bone remodeling and osteoclast differentiation [13, 14]. Siglec-15 exhibits high homology (≥ 30%) with the B7 family and sustainably inhibits the proliferation and activation of T cells independent of the PD-1/PD-L1 pathway [15]. Siglec-15 expression is present in tumor cells (22.8%) and tumor-associated stromal cells (13.3%) in NSCLC. Simultaneously, Siglec-15 has a mutually exclusive expression pattern with PD-L1 [15], suggesting that targeting Siglec-15 may be a novel therapeutic option for patients who are non-responders or resistant to anti-PD-1/PD-L1 immunotherapy [16–19]. The results of a Phase I clinical trial targeting Siglec-15 demonstrate that the anti-Siglec-15 mAb NC318 (NCT03665285) has showed promising clinical efficacy in advanced NSCLC [18, 19]. Moreover, a Phase II clinical trial is ongoing to evaluate its therapeutic efficacy in various advanced or metastatic solid tumors, including lung, breast, uterine, and head and neck cancers [18, 19].

Siglec-15 has received widespread attention over the past 4 years as a novel candidate for cancer immunotherapy normalization strategies [20–26]. We have conducted an integrative pan-cancer analysis of Siglec-15 in public databases [21]. However, it has not yet been systematically investigated in lung adenocarcinoma (LUAD). Therefore, this study comprehensively characterized Siglec-15 expression, highlighted their clinical significance, elucidated the spatial distribution relationship between tumor-infiltrating immune cells (TIICs) and Siglec-15, and provided new insights into anti-Siglec-15 immunotherapy through immunophenotyping based on Siglec-15 expression and CD8A expression/CD8^+^ T cells infiltration. This study aimed to reveal the unique features of Siglec-15 in related to immunosuppressive TIME, and tried to explore specific patient cohorts who most likely to respond to anti-Siglec-15 therapy.

## Materials and methods

### Patient information in the tissue microarray (TMA) of training and validation cohorts

A total of 213 patients with primary LUAD were retrospectively collected as the training cohort, receiving R0 resection from February 2013 to December 2014 at Tianjin Medical University Cancer Institute and Hospital (TMUCIH) in PANEL-1 (Additional file 1: Table S1). Among them, 196 patients with complete OS and disease-free survival (DFS) data were used for the survival analysis. In addition, we collected Xinchao cohort 04 and 07 as validation cohorts, which were purchased from Shanghai Outdo Biotech (Shanghai, China), including LUAD samples of 83 and 68 patients, respectively (Additional file 1: Table S1). All data and images shown in this paper were obtained using TMA. Patient inclusion criteria and TMA construction were shown in Additional file 1.

### Multiplex fluorescence‐based immunohistochemistry (*mfIHC*) and multispectral imaging

All data and images shown in this paper were conducted by *mfIHC*, except for those in Additional file 1: Figure S1. This staining was performed based on the manufacturer’s protocol (PerkinElmer, Opal® Kit) to visualize 8 specific cell markers (Additional file 1: Table S2). The specific experimental operation steps are reflected in Additional file 1.

The stained FFPE tissue sections were scanned using a Vectra microscope. Next, regions of interest (ROIs) were selected with fixed-size stamps (931 × 698 µm; 20× object lens) in Phenochart (PerkinElmer), based on the acquired whole slide scan images. Six filter cubes were used for each image capture, including DAPI (368–461 nm), 480 (450–500 nm), FITC (494–536 nm), CY3 (550–570 nm), CY5 (627–694 nm), and Texas Red (588–616 nm). Three ROIs of 0.65 mm^2^ were selected for each tumor core in order to cover the entire tumor core as much as possible, then each ROIs were scanned at 200× magnification using a Ventana Image Viewer with the same exposure times.

### Spectral unmixing and phenotyping

Multispectral images unmixing was performed using PerkinElmer inForm Image Analysis software (version 2.6.0). We divided the total tissue into tumor area and stromal area based on Pan-cytokeratin (CK) staining. Cells were phenotyped into different classes in PANEL-2 (n = 189), according to our markers of interest as follows: tumor cells (Pan-CK^+^), stroma cells (Pan-CK^−^), T helper cells (CD4^+^), cytotoxic T cells (CD8^+^), effector T cells (Teffs) (CD4^+^FoxP3^−^), T regulatory cells (Tregs) (CD4^+^FoxP3^+^), tumor-associated macrophages (TAMs) (CD68^+^), M2 like TAMs (CD68^+^CD163^+^). Sigelc-15 expression was quantified as percentage on tumor cells or TAMs. The infiltration level of TIICs was quantified as density of cells per mm^2^ in total, tumor, and stroma areas. Differential analysis related to immune cells was plotted as the box plot by https://hiplot.com.cn.

### Spatial analysis of cell phenotypic data

Each image with phenotyped cells was considered as a bivariate planar marked point pattern. Distance between two cells was calculated using the x and y coordinates from the inForm raw data. Using the Euclidean distance formula, each cell of the same phenotype can be used as a reference cell to calculate its distance to other cell of different phenotypes. For each image, the spatial density is the number of other cell type within a given radius around the reference cell, and normalized to the area of tissue (mm^2^). The spatial proximity distance was the mean distance from the reference cell to other cells of different phenotype, within a given radius of the reference cell.

### Gene set variation analysis (GSVA)

FPKM data of LUAD were downloaded from TCGA database (https://www.cancer.gov/tcga/) [27], and then were converted to Log_2_ (FPKM + 1). Gene sets were downloaded from MSigDB database (https://www.gsea-msigdb.org/gsea/msigdb) [28], and the results of Gene Set Variation Analysis (GSVA) were analyzed by “limma” package to identify significantly different gene sets between samples [29]. The bar chart was plotted by https://www.bioinformatics.com.cn.

### Immunotherapy predictors/signatures analysis

TMB data were obtained from Thorsson et al. [30], and MSI data from Russell et al. [31]. Tumor immune dysfunction and exclusion (TIDE) was obtained by submitting transcriptomic data of LUAD through TIDE website (http://tide.dfci.harvard.edu/login/) [32]. The immunophenotype score (Immunophenoscore, IPS) was available via TCIA website (https://tcia.at/tools/toolsMain) [33]. The violin plot was plotted by https://hiplot.com.cn.

### Statistical analysis

GraphPad Prism version 9.0 was used for graph drawing and statistical analyses. Additionally, using five cut-offs (optimal, ≥ 1%, ≥ 5%, and ≥ 25%, we respectively determined the distribution of Sigelc-15. Patients were classified as “Siglec-15 Low” and “Siglec-15 High” groups according to Youden index to achieve the optimal cut-offs. Kaplan–Meier curves were used and estimated by the log-rank test by R version 4.1.2 using survival package. Univariate and multivariate regression analyses was performed by Cox regression analysis by SPSS statistical software (version 26). Comparisons were performed using Wilcoxon test, Kruskal–Wallis test, and Chi-square test as appropriate. All statistics in association between Siglec-15 and clinical parameters were two-sided and analyzed through SPSS statistical software (version 26). Two-sided *P*-values less than 0.05 were considered significant.

## Results

### Expression signature of Siglec-15 in the tumor and macrophage compartment in LUAD in TMUCIH cohort (n = 213)

**Fig. 1:**
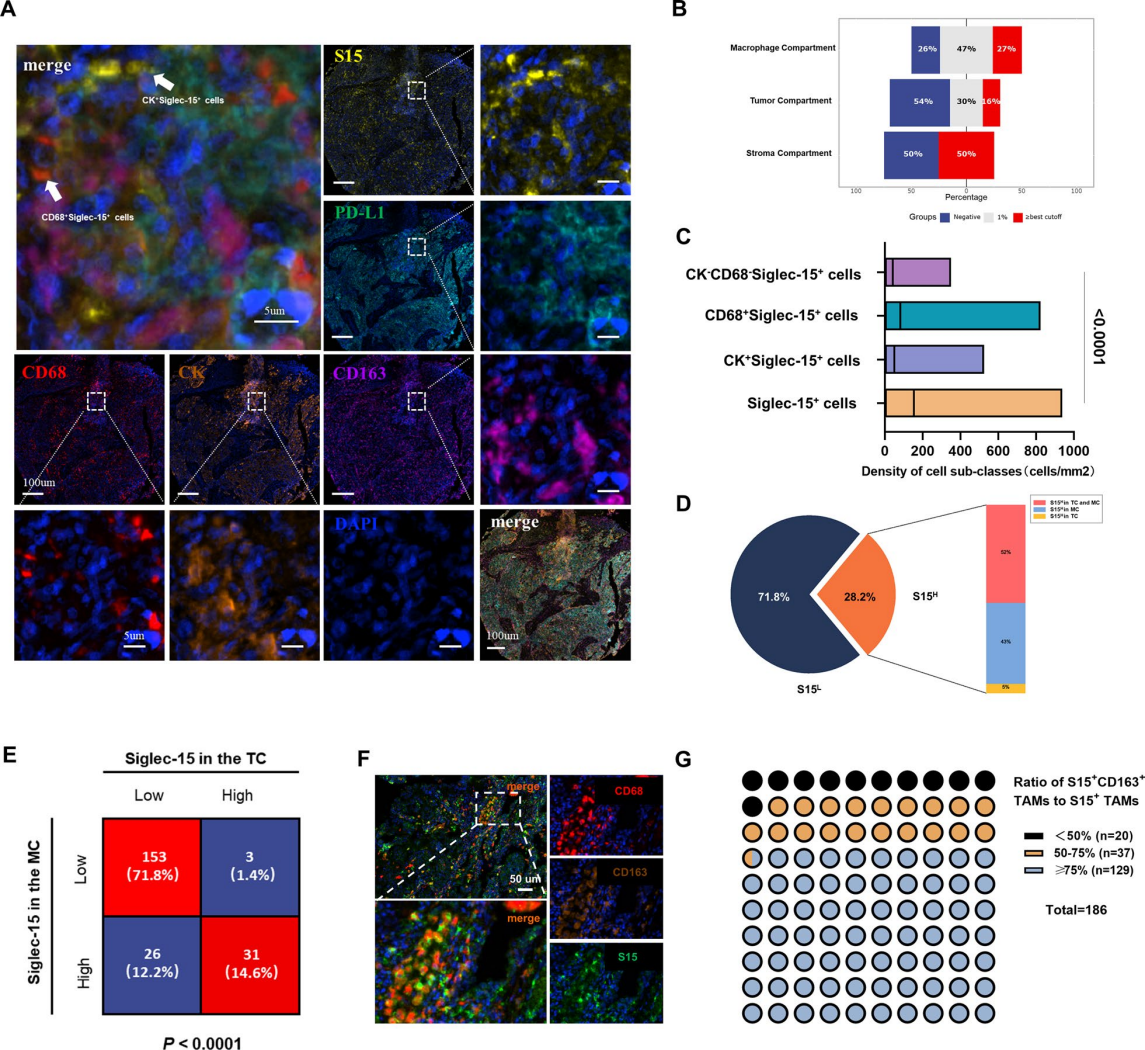
Expression signature of Siglec-15 in the tumor and macrophage compartment in LUAD in TMUCIH cohort (n = 213). **A** Representative *mfIHC* images displayed Siglec-15 expression in LUAD. Scale bar, 5 μm. **B** Positivity rate for Siglec-15 expression was 46% in the TC, 74% in the MC, and 50% in the SC, at a cut-off value of 1%. **C** Siglec-15 expression was higher in CK^+^ cells and CD68^+^ cells than that in the CK^−^CD68^−^ cells. **D** 28.2% of patients with LUAD displayed S15^H^. **E** Siglec15 expression proportion in tumor and macrophage. **F** Representative *mfIHC* images showed Siglec-15 was predominantly expressed in CD163^+^ M2-like TAMs. Scale bar, 50 μm. **G** Siglec-15 was predominantly expressed in M2-like TAMs

It has been reported that Siglec-15 expression is highly upregulated on tumor cells and tumor-infiltrating myeloid cells [15]. However, no further research is known about the expression pattern of Siglec-15 expression. Firstly, Siglec-15 expression were detected by IHC in LUAD in TMUCIH cohort (Additional file 1: Figure S1). Then Siglec-15 expression in the tumor and macrophage compartment were analyzed separately by mfIHC (Fig. 1A), in which CK^+^ cells were defined as the tumor compartment (TC), CD68^+^ cells as the macrophage compartment (MC), and CK^−^ cells as the stroma compartment (SC). The positivity rate of Siglec-15 expression was 46% in the TC, 74% in the MC, and 50% in the SC, with a cut-off value of 1% (Fig. 1B). Siglec-15 expression was higher in CK^+^ cells and CD68^+^ cells than that in the CK^−^CD68^−^ cells (Fig. 1C). 28.2% patients displayed high Siglec-15 expression (S15^H^), of which 52% simultaneously displayed S15^H^ in both the TC and MC, 43% displayed S15-H only in the MC, and 5% displayed S15^H^ only in the TC (Fig. 1D). Totally, 14.6% patients had S15^H^ both in the TC and MC, 1.4% patients had S15^H^ only in the TC, 12.2% patients had S15^H^ only in the MC, 71.8% patients had low Siglec-15 expression (S15^L^) in the TC and MC, respectively (Fig. 1E). Next, the ratio of S15^+^ M2-like TAMs among S15^+^ TAMs were identified (Fig. 1F). The results indicated that 10.7% patients were with low (< 50%), 19.9% patients with intermediate (50–75%), and 69.3% patients with high S15^+^CD163^+^ TAMs expression rate (≥ 75%) (Fig. 1G). Together, 89.2% patients showed intermediate and high S15^+^CD163^+^ TAMs expression ratio, which confirmed that Siglec-15 was predominantly expressed in M2-like TAMs in LUAD (Additional file 1: Figure S2A–E).

Accordingly, these results indicated that Siglec-15 was primarily expressed on tumor cells and M2-like TAMs, and Siglec-15 expressed in the MC showed a higher positivity rate compared to that in the TC.

### Siglec-15 expression in the tumor and macrophage compartment were both associated with poor prognosis in LUAD (n = 196)

**Fig. 2:**
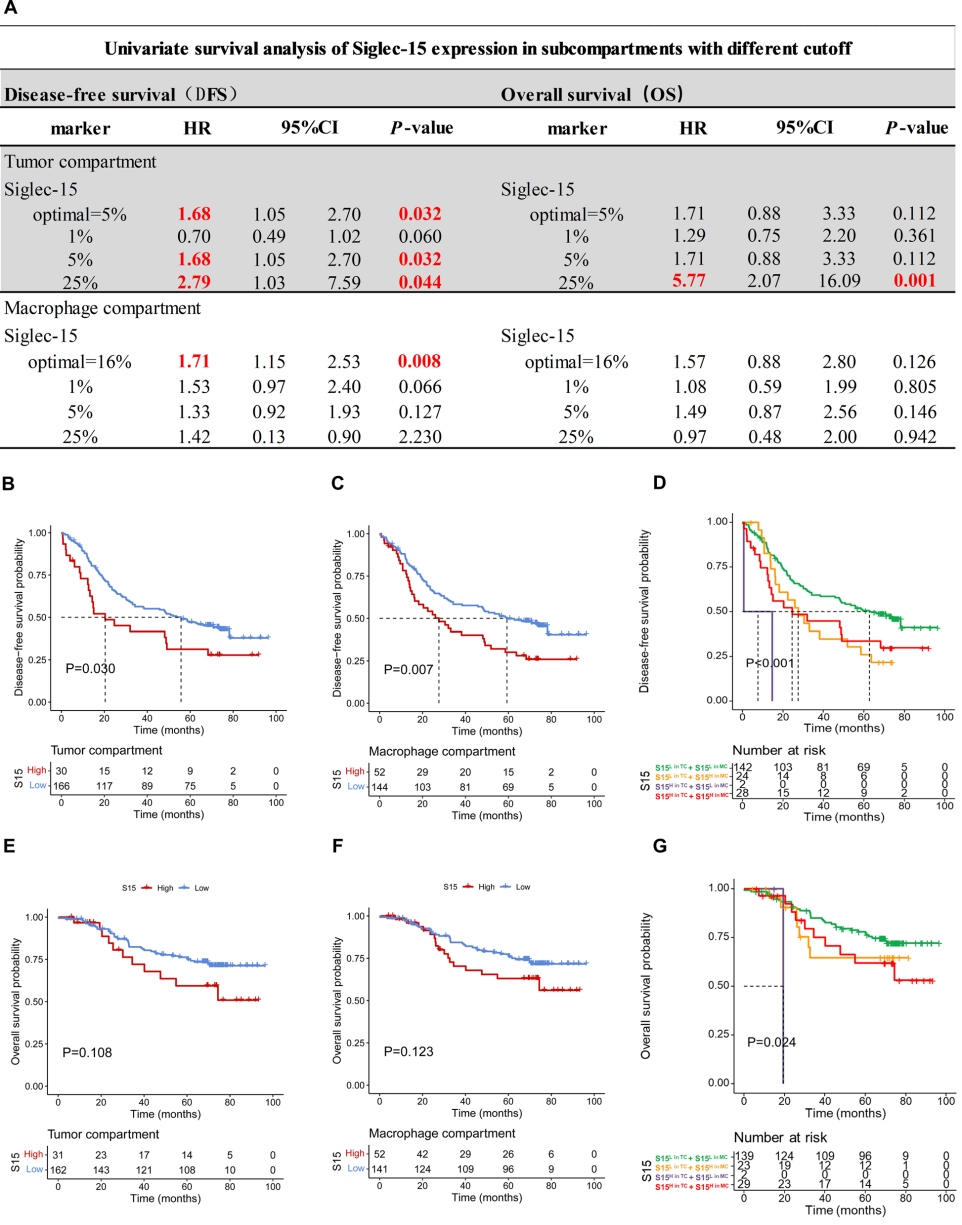
The prognosis value of Siglec-15 in LUAD in TMUCIH cohort (n = 196). **A** Univariate survival analysis of Siglec-15 expression in the tumor and macrophage compartments with different cut-off. **B** S15^H^ in the TC were associated with shorter DFS. **C** S15^H^ in the MC were associated with shorter DFS. **D** Patients with S15^L^ both in the TC and MC showed the best prognosis in DFS, with S15^H^ in the TC or/and MC showed worse prognosis. **E** S15^H^ in the TC had no relationship with OS. **F** S15^H^ had no relationship with OS. **G** Patients with S15^L^ both in the TC and MC showed the best prognosis in OS, with S15^H^ in the TC or/and MC showed worse prognosis

To further identify whether Siglec-15 expression in the TC and MC has the specific clinical values, firstly, univariate Cox regression analysis demonstrated that Siglec-15 had prognostic impact at different cut-off values (Fig. 2A). Then the optimal cut-off values were chosen to define S15^H^ in the TC (cut-off value = 5%), and MC (cut-off value = 16%). Kaplan–Meier analysis results revealed that S15^H^ in the TC (Fig. 2B), and MC (Fig. 2C) were associated with shorter DFS. Based on the cut-off value, no significant effect on LUAD OS was observed in patients (Fig. 2E, F). The results from external validation performed on two other LUAD cohorts also showed that S15^H^ predicted worse prognosis (Additional file 1: Figure S2F–I). Besides, patients with S15^L^ both in the TC and MC showed the best prognosis in DFS (Fig. 2D), and OS (Fig. 2G), while patient with S15^H^ in the TC or / and MC showed worse prognosis. Furthermore, the univariate and multivariate Cox regression analysis of Siglec-15 were systematically investigated. The results identified the T classification, N classification, and Siglec-15 expression in the MC, but not in the TC, as independent prognostic markers for DFS (Table 1). In addition, there was no association between Siglec-15 expression and clinicopathological parameters in the TC (Additional file 1: Table S3), or MC (Additional file 1: Table S4).

**Table 1:** Univariate and Multivariate analysis of Siglec-15 and clinicopathological factors in LUAD in TMUCIH cohort

Variables	Univariate analysis		Multivariate analysis			
			S15 in TC		S15 in MC	
	n	*P*	HR (95%CI)	*P*	HR (95%CI)	*P*
Gender						
Females	114	0.339	–	–	–	–
Males	82					
Age (years)						
≥ 60	92	0.568	–	–	–	–
< 60	104					
Smoking						
Yes	69	0.686	–	–	–	–
No	127					
T classification						
T_2–4_	94	**< 0.001**	1.577 (1.057–2.354)	**0.026**	1.570 (1.052–2.342)	**0.027**
T_1_	102					
N classification						
N_1–2_	72	**< 0.001**	2.179 (1.452–3.271)	**< 0.001**	2.163 (1.445–3.238)	**< 0.001**
N_0_	124					
S15 in the TC						
High	30	**0.032**	1.389 (0.857–2.252)	0.183		
Low	166					
S15 in the MC						
High	144	**0.008**			1.497 (1.006–2.228)	**0.047**
Low	52					

Statistically significant differences (*P* < 0.05) are bolded

Multivariate analysis was performed by the Cox multivariate proportional hazard regression model with stepwise manner

TNM Tumor-nodes-metastases, HR Hazard ratio, CI Confidential interval, S15 Siglec-15

Therefore, these results revealed that Siglec-15 in the TC or MC were both associated with poor prognosis for DFS in LUAD, and S15^H^ in the MC have greater prognostic value than that in the TC.

### Siglec-15 expression in the tumor and macrophage compartment were both accompanied with immunosuppressive landscape (n = 189)

**Fig. 3:**
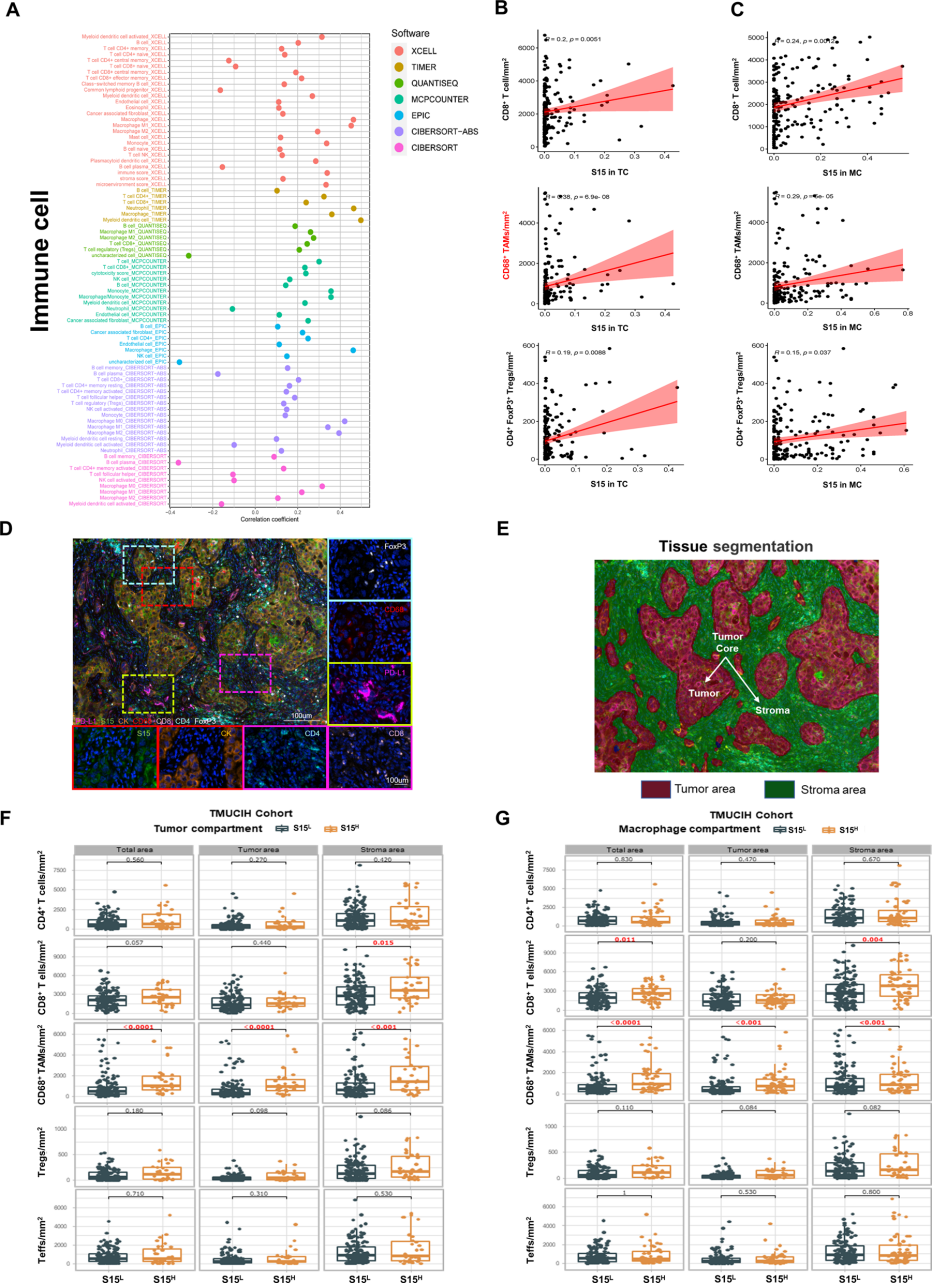
Relationship between Siglec-15 and TIICs in LUAD in TMUCIH cohort (n = 189). **A** In TCGA database, Siglec-15 was positively correlated with TAMs and Tregs. **B** Siglec-15 in the TC was positively correlated with the density of CD8^+^ T cells, CD68^+^ TAMs, and CD4^+^FoxP3^+^ Tregs. **C** Siglec-15 in the MC was positively correlated with the density of CD8^+^ T cells, CD68^+^ TAMs, and CD4^+^FoxP3^+^ Tregs. **D** Representative *mfIHC* images of Siglec-15 and basic immune cell landscape in LUAD. **E** Based on tissue segmentation, the density of each immune cell population was calculated in the total, tumor, and stroma areas. **F** Patients with S15^H^ (n = 32) in the TC had more CD8^+^ T cells than those with S15^L^ (n = 157) in the stroma area, more CD68^+^ TAMs than those with S15^L^ in the total, tumor, or stroma areas. **G** Patients with S15^H^ (n = 53) in the MC had more CD8^+^ T cells than those with S15^L^ (n = 136) in the total and stroma areas, more CD68^+^ TAMs than those with S15^L^ in the total, tumor, or stroma areas

The TIME complex is closely related to tumor progression and immunotherapy efficacy [34, 35]. Therefore, it is important to explore the relationship between Siglec-15 and TIICs. In TCGA, Siglec-15 was positively correlated with TAMs and Tregs, which were analyzed by 7 algorithms (Fig. 3A). Meanwhile, CD8^+^ T cells, CD4^+^ T cells, CD4^+^FoxP3^+^ Tregs, CD4^+^FoxP3^−^ Teffs, and CD68^+^ TAMs were analyzed in the total, tumor, and stroma area, respectively (Fig. 3D, E). Siglec-15 expression in the TC was significantly positively correlated with the infiltration of TAMs (*r* = 0.38), CD8^+^ T cells (*r* = 0.20), and Tregs (*r* = 0.19) (Fig. 3B). Siglec-15 expression in the MC was significantly positively correlated with the infiltration of TAMs (*r* = 0.29), CD8^+^ cells (*r* = 0.24), and Tregs (*r* = 0.15) (Fig. 3C). No significant correlation was observed for the infiltration of CD4^+^ T cells and Teffs (Additional file 1: Figure S3A, B).

It was worth mentioning that, in the TC, patients with S15^H^ (n = 32) had higher infiltration of CD8^+^ T cells than those with S15^L^ (n = 157) only in the stroma area (*P* = 0.015), and had higher infiltration of TAMs than those with S15^L^ in the total (*P* < 0.0001), tumor (*P* < 0.0001), or stroma area (*P* < 0.001) (Fig. 3F). In the MC, patients with S15^H^ (n = 53) had higher infiltration of CD8^+^ T cells than those with S15^L^ (n = 136) in the total (*P* = 0.011), or stroma areas (*P* = 0.004), whereas patients with S15^H^ had higher infiltration of TAMs than those with S15^L^ in the total (*P* < 0.0001), tumor (*P* < 0.001), or stroma area (*P* < 0.001) (Fig. 3G). There were no significant differences in the infiltration of CD4^+^ T cells, Tregs, and Teffs between the S15^H^ and S15^L^ groups. Moreover, the above results were confirmed in another cohort (Additional file 1: Figure S3C, D).

Taken together, although Siglec-15 expression was positively correlated with the infiltration of CD8^+^ T cells, CD8^+^ T cells were mostly infiltrated in the stroma area, not in the tumor area, which indicated that Siglec-15 was overexpressed in an immune-excluded LUAD TIME [36, 37]. Furthermore, S15^H^ was accompanied with more TAMs and Tregs infiltration, which indicated an immunosuppressive microenvironment in LUAD.

### Siglec-15^+^ TAMs were more closely related to CD8^+^ T cells spatial distribution in PD-L1^−^ cells (n = 189)

**Fig. 4:**
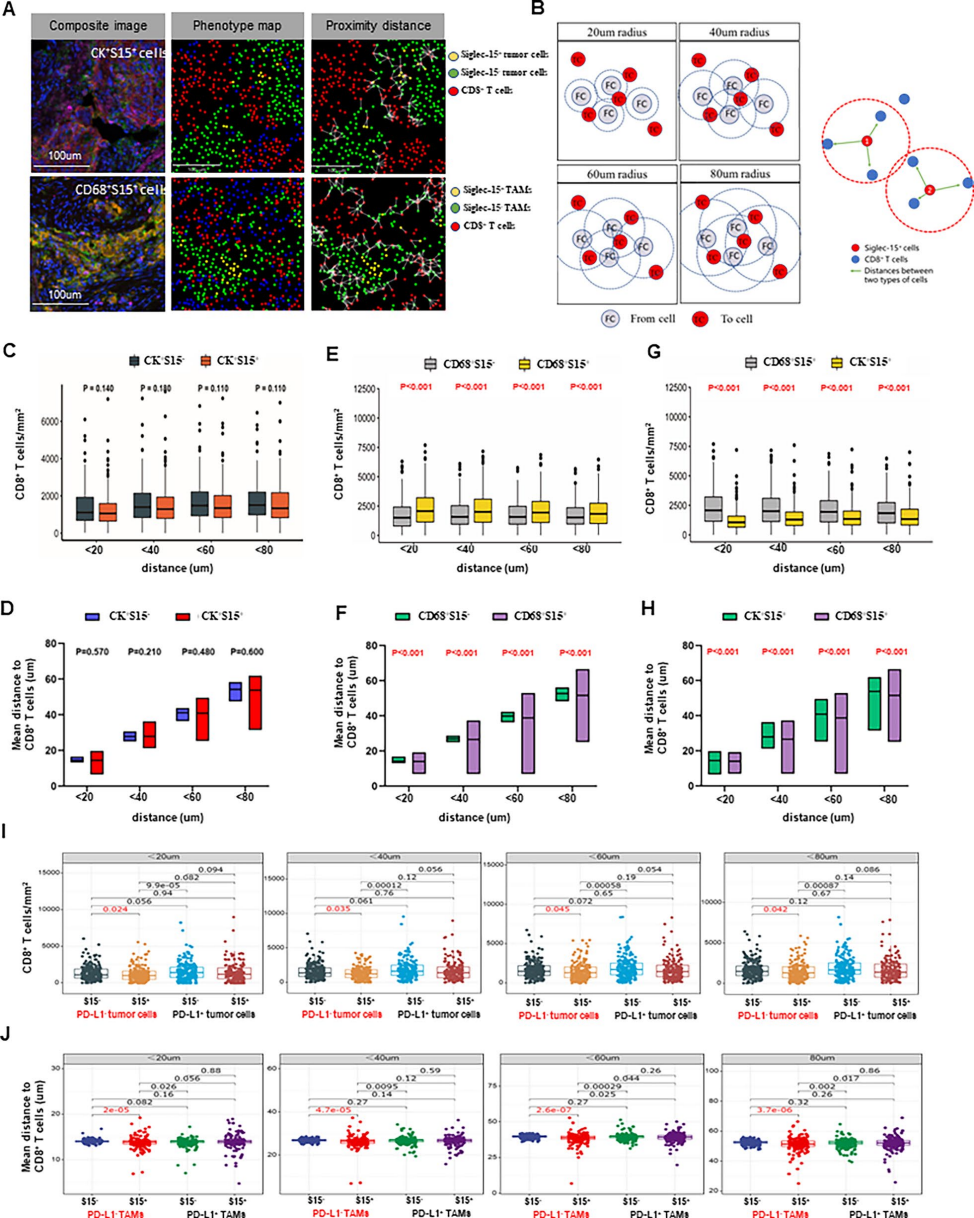
Siglec-15^+^ TAMs were more closely related to CD8^+^ T cells spatial distribution in PD-L1^−^ cells (n = 189). **A** Representative composite image, phenotype map, and proximity distance map showing CD8^+^ T cells within a 20um radius from the nuclear center of each S15^−^ and S15^+^ tumor cells or TAMs. Scale bar, 100 um. **B** Accurate spatial analysis over multiple distance ranges (< 20, 40, 60, or 80 μm) in terms of spatial density and distance. **C** There was no significant difference in the spatial density of CD8^+^ T cells, (**D**) and distance to CD8^+^ T cells between S15^+^ and S15^−^ tumor cells. **E** The density of CD8^+^ T cells around S15^+^ TAMs were significantly higher than that around both S15^−^ TAMs, (**G**) and S15^+^ tumor cells. **F** S15^+^ TAMs were spatially closer to CD8^+^ T cells than S15^−^ TAMs, (**H**) and S15^+^ tumor cells. **I** Lower infiltrating CD8^+^ T cells surrounding PD-L1^−^ + S15^+^ tumor cells than PD-L1^−^ + S15^−^ tumor cells. **J** PD-L1^−^ + S15^+^ TAMs were significantly closer proximity to CD8^+^ T cells than PD-L1^−^ + S15^−^ TAMs

Combined with the above results, the relationship between Siglec-15 and CD8^+^ T cells were further studied from the perspective of single cell using spatial analysis. The spatial density of CD8^+^ T cells around S15^+^ and S15^−^ tumor cells or TAMs, and the proximity distance from these cells to CD8^+^ T cells were recorded (Fig. 4A). An accurate analysis was carried out over multiple distance ranges (< 20, 40, 60, or 80 μm) (Fig. 4B). There was no significant difference in the density of CD8^+^ T cells infiltration between S15^+^ and S15^−^ tumor cells (Fig. 4C), and in the distance from these cells to CD8^+^ T cells (Fig. 4D). However, heterogeneous cellular spatial patterns of CD8^+^ T cells around S15^+^ and S15^−^ TAMs were showed. The density of CD8^+^ T cells infiltrating around S15^+^ TAMs was significantly higher than that around both S15^−^ TAMs (Fig. 4E), and S15^+^ tumor cells (Fig. 4G). Besides, S15^+^ TAMs were spatially closer to CD8^+^ T cells than both S15^−^ TAMs (Fig. 4F), and S15^+^ tumor cells (Fig. 4H). These findings suggested that S15^+^ TAMs are more likely to directly interact with CD8^+^ T cells than S15^−^ TAMs and S15^+^ tumor cells.

Considering Siglec-15 and PD-L1 expression characteristics, the spatial distribution of CD8^+^ T cells were then compared simultaneously. In the PD-L1-negative (PD-L1^−^) tumor cells, fewer infiltrating CD8^+^ T cells were surrounding PD-L1-negative and S15-positive (PD-L1^−^ + S15^+^) tumor cells than PD-L1-negative and S15-negative (PD-L1^−^ + S15^−^) tumor cells in different ranges (Fig. 4I). Spatial proximity distance analysis revealed that PD-L1^−^ + S15^+^ tumor cells showed slightly farther distance to CD8^+^ T cells than PD-L1^−^ + S15^−^ tumor cell at a distance of less than 40 um (Additional file 1: Figure S4A). In the PD-L1^−^ TAMs, more CD8^+^ T cells were infiltrated surrounding PD-L1^−^ + S15^+^ TAMs than PD-L1^−^ + S15^−^ TAMs, only in the range of less than 20, 40 um (Additional file 1: Figure S4B). PD-L1^−^ + S15^+^ TAMs were significantly closer proximity to CD8^+^ T cells than PD-L1^−^ + S15^−^ TAMs in different ranges (Fig. 4J). In the PD-L1^+^ cells, there was no difference, which indicated that PD-L1 may affect the spatial distribution of CD8^+^ T cells around Siglec-15^+^ tumor cells or TAMs.

Collectively, S15^+^ tumor cells had fewer CD8^+^ T cells density, and S15^+^ TAMs were spatially closer to CD8^+^ T cells in the PD-L1^−^ cells, which indicated the difference between S15^+^ tumor cells and S15^+^ TAMs in relation to CD8^+^ T cells.

### Siglec-15^+^ tumor cells or TAMs were strongly associated with the spatial distribution of Tregs and TAMs (n = 189)

**Fig. 5:**
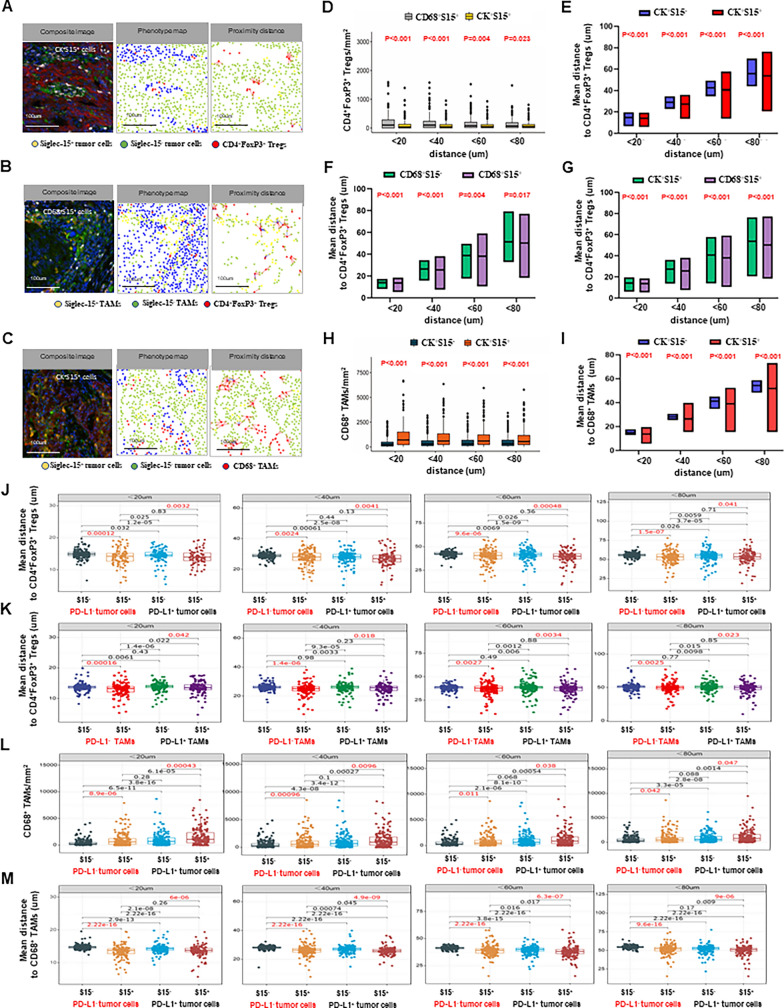
Siglec-15^+^ tumor cells or TAMs were strongly associated with the spatial distribution of Tregs and TAMs (n = 189). **A** Representative composite image, phenotype map, proximity distance map showing CD4^+^FoxP3^+^ Tregs within a 20um radius from the nuclear center of each S15^−^ and S15^+^ tumor cells, (**B**) or TAMs, (**C**) and CD68^+^ TAMs within a 20um radius from the nuclear center of each S15^−^ and S15^+^ tumor cells. Scale bar, 100 um. **D** The CD4^+^FoxP3^+^ Tregs density around S15^+^ TAMs was significantly higher than that around S15^+^ tumor cells. **E** S15^+^ tumor cells were closer proximity to CD4^+^FoxP3^+^ Tregs compared with S15^−^ tumor cells. **F** S15^+^ TAMs were spatially closer to CD4^+^FoxP3^+^ Tregs than S15^−^ TAMs, (**G**) and S15^+^ tumor cells. **J** S15^+^ tumor cells or TAMs were spatially closer to CD4^+^FoxP3^+^ Tregs than S15^−^ tumor cells, (**K**) and spatially closer to CD4^+^FoxP3^+^ Tregs than S15^−^ TAMs, regardless of PD-L1 expression. **H** S15^+^ tumor cells were surrounded by more CD68^+^ TAMs, (**I**) and spatially closer to CD68^+^ TAMs than S15^−^ tumor cells. **L** S15^+^ tumor cells were infiltrated by more CD68^+^ TAMs than S15^−^ tumor cells, (**M**) and were spatially closer to CD68^+^ TAMs than S15^−^ tumor cells, regardless of PD-L1 expression

Similarly, spatial density and proximity distance of CD4^+^FoxP3^+^ Tregs and CD68^+^ TAMs (Fig. 5A–C) were analyzed. No difference in CD4^+^FoxP3^+^ Tregs density was observed between S15^−^ and S15^+^ tumor cells (Additional file 1: Figure S4C, E) or TAMs (Additional file 1: Figure S4D, F). However, the CD4^+^FoxP3^+^ Tregs density around S15^+^ TAMs was significantly higher than that around S15^+^ tumor cells (Fig. 5D). It is worth noting that S15^+^ tumor cells were closer proximity to CD4^+^FoxP3^+^ Tregs compared with S15^−^ tumor cells (Fig. 5E). However, S15^+^ TAMs were spatially closer to CD4^+^FoxP3^+^ Tregs than S15^−^ TAMs (Fig. 5F), and S15^+^ tumor cells (Fig. 5G) in distinct ranges. S15^+^ tumor cells or TAMs were spatially closer to CD4^+^FoxP3^+^ Tregs than S15^−^ tumor cells or TAMs, both in the PD-L1^−^ or PD-L1^+^ tumor cells (Fig. 5J) or TAMs (Fig. 5K).

In addition, in the range of less than 20 um to 80 um, S15^+^ tumor cells were surrounded by more CD68^+^ TAMs (Fig. 5H), and spatially closer to CD68^+^ TAMs than S15^−^ tumor cells (Fig. 5I). No matter PD-L1 was present or not, S15^+^ tumor cells were infiltrated by more CD68^+^ TAMs (Fig. 5L), and closer to CD68^+^ TAMs than S15^−^ tumor cells (Fig. 5M).

Summarily, S15^+^ tumor cells or TAMs were spatially closer to CD4^+^FoxP3^+^ Tregs, and S15^+^ tumor cells were positively correlated with the spatial density of TAMs, and spatially closer to TAMs.

### Siglec-15^+^ tumor cells or TAMs reversed CD8^+^ T cells prognosis value, and enhanced the prognosis value of Tregs and TAMs (n = 189)

**Fig. 6:**
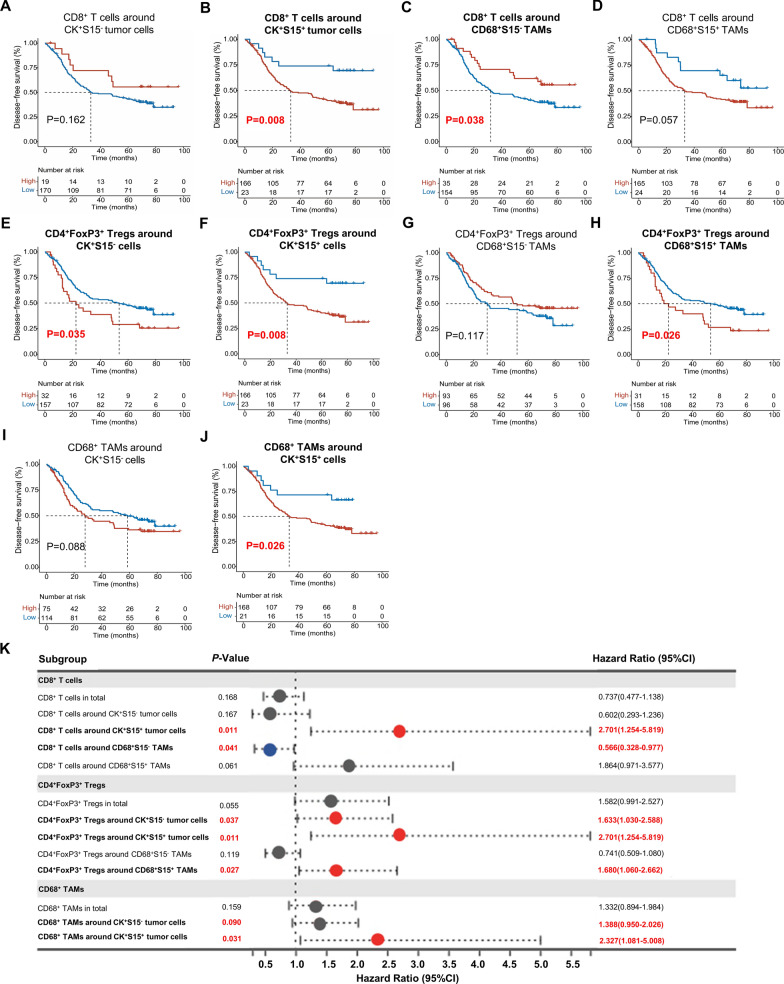
Siglec-15^+^ tumor cells or TAMs reversed CD8^+^ T cells prognosis value, and enhanced the prognosis value of Tregs and TAMs. **A** Kaplan–Meier survival analysis showed that CD8^+^ T cells localized to S15^−^ tumor cells had no prognostic significance (*P* = 0.162). **B** CD8^+^ T cells localized to S15^+^ tumor cells had an adverse effect on prognosis (*P* = 0.008). **C** CD8^+^ T cells predominantly localized to S15^−^ TAMs demonstrated a favorable effect on prognosis (*P* = 0.038). **D** CD8^+^ T cells predominantly localized to S15^+ ^TAMs tend to have an adverse effect on prognosis (*P* = 0.057). **E** CD4^+^FoxP3^+^ Tregs surrounding S15^−^ tumor cells were associated with a bad prognosis (*P* = 0.035). **F** CD4^+^FoxP3^+^ Tregs surrounding S15^+^ tumor cells were correlated with poor outcome (*P* = 0.008). **G** CD4^+^FoxP3^+^ Tregs predominantly localized to S15^−^ TAMs had no effect on patient's survival (*P* = 0.117). **H** CD4^+^FoxP3^+^ Tregs predominantly localized to S15^+^ TAMs had an adverse effect on prognosis (*P* = 0.026). **I** CD68^+^ TAMs surrounding S15^−^ tumor cells had no significant effect on the prognosis (*P* = 0.088). **J** CD68^+^ TAMs predominantly localized to S15^+^ tumor cells demonstrated an adverse effect on prognosis (*P* = 0.026). **K** Univariate Cox regression analysis demonstrated that Siglec-15 do work as a risk factor associated with the prognosis of these immune cells

In addition, CD8^+^ T cells prognostic value were altered by Siglec-15. CD8^+^ T cells had no significant effect on patient survival in the TME (*P* = 0.167) (Additional file 1: Figure S4G). The prognostic value of CD8^+^ T cells were diametrically opposed around S15^+^ and S15^−^ tumor cells or TAMs (Fig. 6A–D). CD8^+^ T cells localized to S15^−^ tumor cells had no prognostic significance (*P* = 0.162) (Fig. 6A). It was worth noting that, CD8^+^ T cells predominantly localized to S15^+^ tumor cells demonstrated an adverse effect on prognosis (*P* = 0.008) (Fig. 6B). CD8^+^ T cells predominantly localized to S15^−^ TAMs demonstrated a favorable effect on prognosis (*P* = 0.038) (Fig. 6C). Whereas CD8^+^ T cells predominantly localized to S15^+^ TAMs tended to have an adverse effect on prognosis although the statistical significance was not significant (*P* = 0.057) (Fig. 6D). Furthermore, these findings reaffirmed the inhibitory effect of Siglec-15 on T cells.

CD4^+^FoxP3^+^ Tregs were associated with a bad prognosis in the TME (*P* = 0.044) (Additional file 1: Figure S4H), as well as CD4^+^FoxP3^+^ Tregs surrounding S15^−^ tumor cells (*P* = 0.035) (Fig. 6E), and S15^+^ tumor cells (*P* = 0.008) (Fig. 6F). It is worth mentioning that CD4^+^FoxP3^+^ Tregs surrounding S15^+^ tumor cells had the most significant prognostic values (*P* = 0.008) (Fig. 6F). CD4^+^FoxP3^+^ Tregs had no effect on patient's survival when Tregs were predominantly localized to S15^−^ TAMs (*P* = 0.117) (Fig. 6G). Whereas CD4^+^FoxP3^+^ Tregs demonstrated an adverse effect on prognosis when CD4^+^FoxP3^+^ Tregs were predominantly localized to S15^+^ TAMs (*P* = 0.026) (Fig. 6H). These results also suggested that the presence of Siglec-15 may enhance the status of Tregs as adverse prognostic factors.

CD68^+^ TAMs had no prognostic significance (*P* = 0.158) (Additional file 1: Figure S4I), as well as CD68^+^ TAMs surrounding S15^−^ tumor cells (*P* = 0.088) (Fig. 6I). Notably, CD68^+^ TAMs predominantly localized to S15^+^ tumor cells demonstrated an adverse effect on prognosis (*P* = 0.026) (Fig. 6J), suggesting that S15^+^ tumor cells might enhance the immunosuppressive role of CD68^+^ TAMs. Finally, univariate Cox regression analysis further demonstrated that Siglec-15 functions as a risk factor associated with the prognosis of these immune cells (Fig. 6K).

In general, S15^+^ tumor cells or TAMs reversed CD8^+^ T cells prognosis value. Additionally, they enhanced CD4^+^FoxP3^+^ Tregs and TAMs prognosis value.

### Tumor microenvironment immune type (TMIT) based on Siglec-15 and CD8A expression in TCGA

**Fig. 7:**
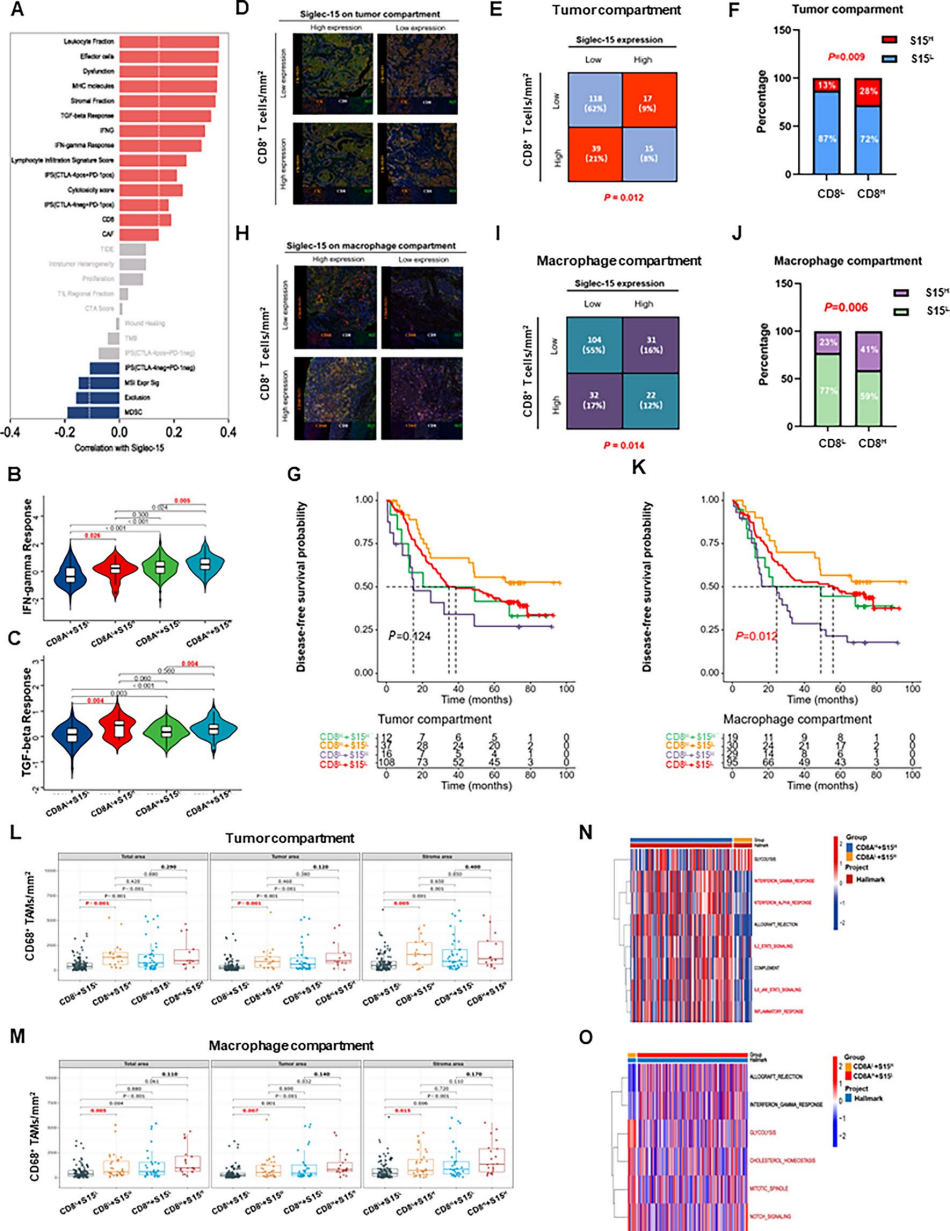
Tumor microenvironment immune type (TMIT) based on Siglec-15 and CD8A/CD8^+^ T cells. **A** Siglec-15 were weakly positively correlated with immune response-related biomarkers and signatures. **B** S15^H^ was accompanied by IFN-γ, (**C**) and TGF-β response activation. **D** Representative *mfIHC* images showed immunotyping based on Siglec-15 expression and CD8^+^ T cells in the TC, (H) and MC. **E** Siglec15 expression proportion in the four TMIT in the TC, (**I**) and MC. **F** The proportion of patients with S15^H^ was higher in the CD8^H^ group in the TC, (**J**) and MC. **G** Patients with CD8^L^ + S15^H^ group had the worst DFS prognosis in the TC, (**K**) and MC. **L** In the case of low CD8^+^ T cell infiltration, the CD68^+^ TAMs density tended to be increased in the S15^H^ group compared with the S15^L^ group in the TC, (**M**) and MC. **N** Patients with CD8A^H^ + S15^H^ exhibited the activation of immune-related hallmark markers. **O** Patients with CD8A^L^ + S15^H^ showed the activation of metabolism-related hallmark markers

According to the non-spatial and spatial results, Siglec-15 was associated with an immunosuppressive microenvironment, which cannot be ignored during the process of anti-Siglec-15 therapy. Then, the relationship between Siglec-15 expression and immune response-related biomarkers or signatures was investigated in TCGA. Positive correlations with Siglec-15 expression were noted (Fig. 7A), including positive immunoregulatory signatures, such as leukocyte fraction, effector cells, MHC molecules, and IFN-γ Response, but also negative immunoregulatory signatures, such as dysfunction, and the TGF-β response (*r* > 0.3, *P* < 0.05).

Moreover, in addition to the number of immune cells, their function is also crucial for immunotherapy, especially CD8^+^ T cells [38, 39]. TMIT is a classification recognizing four tumor immunophenotypes based on the presence of intratumoral CD8^+^ TILs and tumoral PD-L1 expression, which could predict the efficacy of immunotherapy to some extent [37]. Here, a novel TMIT was constructed based on Siglec-15 and the CD8A expression in TCGA or the infiltration of CD8^+^ T cells in TMUCIH cohort. In the CD8A high or low expression subgroups, S15^H^ was accompanied by IFN-γ (Fig. 7B), or TGF-β response activation (Fig. 7C). In terms of two subtypes of IPS values, patients with S15^H^ had a lower IPS of CTLA4^neg^ + PD-1^neg^ (Additional file 1: Figure S5A), or CTLA4^pos^ + PD-1^neg^ (Additional file 1: Figure S5B), only in the CD8A high expression subgroups, indicating that the relative probabilities to response to anti-CTLA-4 mAb treatment were lower in the S15^H^ group. No differences were found in the other signatures between groups based on TMIT (Additional file 1: Figure S5C). In TCGA, 23% patients had high CD8A and high S15 expression (CD8A^H^ + S15^H^), 4.2% patients had low CD8A and high S15 expression (CD8A^L^ + S15^H^), 55.6% patients had high CD8A and low S15 expression (CD8A^H^ + S15^L^), and 17.2% patient had low CD8A and low S15 expression (CD8A^L^ + S15^L^) (Additional file 1: Figure S5D). Patients in the CD8A^L^ + S15^H^ group had the worst OS prognosis, while patients in the CD8A^H^ + S15^L^ group had the best OS prognosis (Additional file 1: Figure S5E).

### Tumor microenvironment immune type (TMIT) based on Siglec-15 expression and CD8^+^ T cells in the TMUCIH cohort (n = 189)

In the TMUCIH cohort (Fig. 7D), 8% patients had high CD8^+^ T cell infiltration and high S15 expression (CD8^H^ + S15^H^), 9% had low CD8^+^ T cell infiltration and high S15 expression (CD8^L^ + S15^H^), 21% had high CD8^+^ T cell infiltration and low S15 expression (CD8^H^ + S15^L^), higher percentage of 62% had low CD8^+^ T cell infiltration and low S15 expression (CD8^L^ + S15^L^) in the TC, respectively (Fig. 7E). Compared with the CD8^L^ group, the proportion of patients with S15^H^ was higher in the CD8^H^ group in the TC (Fig. 7F). Patients in the CD8^L^ + S15^H^ group had the worst DFS (Fig. 7G) or OS prognosis (Additional file 1: Figure S5F), while patients in the CD8^H^ + S15^L^ group had the best DFS (Fig. 7G) or OS prognosis (Additional file 1: Figure S5F), although there is no statistical difference. Next, the infiltration levels of immune cells in TMIT groups were further analyzed. In the CD8^L^ group, the density of CD68^+^ TAMs tended to be increased in the S15^H^ group, compared with the S15^L^ group, but these were not obvious in the CD8^H^ group (Fig. 7L). There were no significant differences in the infiltration of CD4^+^ T cells, CD4^+^FoxP3^+^ Tregs, and CD4^+^FoxP3^−^ Teffs between groups (Additional file 1: Figure S5H).

In the MC (Fig. 7H), 12% patients were with CD8^H^ + S15^H^, 16% were with CD8^L^ + S15^H^, 17% were with CD8^H^ + S15^L^, higher percentage of 55% were with CD8^L^ + S15^L^, respectively (Fig. 7I). The proportion of patients with S15^H^ was also higher in the CD8^H^ group than that in the CD8^L^ group (Fig. 7J). Patients in the CD8^L^ + S15^H^ group had the worst DFS prognosis, while patients in the CD8^H^ + S15^L^ group had the best DFS prognosis, significantly (Fig. 7K). However, there were no significant differences in OS among groups (Additional file 1: Figure S5G).In the MC, the density of CD68^+^ TAMs tended to be increased in the CD8^L^ + S15^H^ group than CD8^L^ + S15^L^ group (Fig. 7M), which were consistent with the results above in the TC, and observed in Xinchao cohort 04 (Additional file 1: Figure S5J, K). Similarly, there were no significant differences in other immune cells between groups (Additional file 1: Figure S5I).

To further investigate the potential reasons for the differences in the TMITs, GSVA was performed and the results indicated that patients with CD8^H^ + S15^H^ exhibited the activation of immune-related hallmark markers, such as IFN-γ response, IFN-α response, IL2-STAT5 signaling, IL6-JAK-STAT3 signaling, and inflammatory response (Fig. 7N). Patients with CD8^L^ + S15^H^ showed the activation of metabolism-related hallmark marker, such as glycolysis, cholesterol homeostasis, and NOTCH signaling (Fig. 7N, O).

Summarily, immunosuppressive state and metabolism-related pathway activation co-existed with high expression of Siglec-15, and patients with CD8^L^ + S15^H^ had the worst prognosis and higher TAMs infiltration, which indicated that Siglec-15 may impact tumor progression mainly when CD8^+^ T cell infiltration was low.

## Discussion

Siglec-15 is considered a novel broad-spectrum immunotherapy target [15, 19]. Phase II clinical trial of an anti-Siglec-15 mAb is currently under investigation [18, 19]. The selection criteria for enrolled patients are the key factor to the effectiveness of the drug. Hence, there is an urgent need to identify the type of patients who are most likely to benefit from anti-Siglec-15 therapy, in order to improve the efficacy of subsequent clinical trials. It is worth pointing out that the patients enrolled in phase II clinical trials were all refractory patients with advanced cancer, who had previously received immunotherapy ineffectively or drug resistance. However, in our present study, patients enrolled were early (stage I, II) and locally advanced (IIIa) LUAD patients who can be operated R0, but not inoperable advanced LUAD. So, our research findings can only represent a part of patients, which is actually the limitation of this study. Furthermore, more exploration of Siglec-15 will be crucial in studying immunotherapy-resistance. We believe that the widespread clinical application of immunotherapy, especially PD-1/PD-L1 therapy, will provide us with more opportunity to study the potential value of Siglec-15, and identify effective target groups for anti-Siglec-15 therapy.

As a classic immune checkpoint, PD-L1 provides valuable experience [40–42]. PD-L1 expression is one of the most used biomarkers for predicting immunotherapy efficacy [42]. In this study, at optimal cut-off values, the Siglec-15 expression positivity rates in the TC and MC were calculated to be 16% and 27%, respectively, and the total positivity rate was 28.2%. In the Phase I / II clinical trial of NC318, the objective response rate of patients with NSCLC was 20%, which was slightly lower than the Siglec-15 expression rate defined in this study. Here, the possible causes of this problem were comprehensively explored. Firstly, the actual predictive role of immune checkpoints may be weakened by various factors [42], such as differences in antibody clones, staining platforms, interpretation criteria, and positive thresholds, as well as the challenges posed by differences in the tissue samples obtained, and spatiotemporal heterogeneity. In addition, there are many other immunotherapeutic biomarkers [39, 43, 44], as well as the TMIT [43] which could predict immunotherapeutic efficacy. Among them, Siglec-15 was positively associated with a negative efficacy predictive biomarker, such as TGF-β, but not with positive efficacy predictive biomarkers, such as TMB or MSI (Fig. 7A). Even with CD8A^H^, the TGF-β scores were higher (Fig. 7C), and IPS were lower in the S15^H^ group than those in the S15^L^ group (Additional file 1: Figure S5A, B). Moreover, Siglec-15 expression was positively correlated with CD68^+^ TAMs and CD4^+^FoxP3^+^ Tregs, both of which secrete TGF-β, and thereby promote the formation of an immunosuppressive microenvironment [45]. Therefore, these phenomena imply that it is difficult for anti-Siglec-15 therapies to exert an effective therapeutic outcome, which may be due to the immunosuppressed state. Based on this immune characteristic, it may be the key for improving the efficacy of anti-Siglec-15 therapies, which effectively eliminate the immune suppression status, and restore the original immune state before anti-Siglec-15 treatment.

Moreover, the density of CD8^+^ T cells was significantly increased in the stromal area in the S15^H^ group when compared to that of the S15^L^ group. However, there was no significant difference in CD8^+^ T cell infiltration in the tumor area between the S15^H^ and S15^L^ groups (Fig. 3F, G). These results suggested that although Siglec-15 was highly expressed in LUAD, which was identified as a “hot tumor”, S15^+^ cells is most likely to be predominant in the “immune excluded” region. Moreover, Siglec-15 may have two immune escape mechanisms [46]: direct binding with T cells to inhibit T cell function in cytotoxic T lymphocyte (CTL) infiltration-rich regions, or CTL infiltration inhibition through some yet unclear factors. These mechanisms may be major challenges underlying the use of anti-Siglec-15 therapy. Whether S15^+^ cells could inhibit T cell infiltration or not is required further studies. So, patients with CD8^L^ + S15^H^ were the potential applicable population for anti-Siglec-15 treatment, if combination therapy of relieving patient immune suppression were applied before anti-Siglec-15 therapy, which might potentially maximize anti-Siglec-15 therapeutic efficacy and make Siglec-15 a broad-spectrum immunotherapy target.

It is a big challenge and needs a long way to explore a new immune checkpoint molecule [44, 47–50]. The development of new drugs is not smooth sailing, and the transition from basic research to clinical practice requires a long time. Although, our study exists certain limitations, it may also provide potential value for the determination of indications of Siglec-15 monoclonal antibody, and be beneficial to design clinical trials targeting advanced patients based on molecular characteristics of Siglec-15 in the future. More studies of Siglec-15 are still necessary and hopefully these studies will provide more clues to further clarify the application value of Siglec-15 in the future.

## Conclusions

In summary, Siglec-15 expressed on TAMs showed a higher positivity rate and more important prognostic value than that on tumor cells. Siglec-15 was accompanied by an immunosuppressed state with the lack of intratumoral CD8^+^ T cells infiltration, excessive Tregs and TAMs infiltration, and immunosuppression activation, which is the critical issues cannot be ignored in anti-Siglec-15 therapies. Spatially, S15^+^ tumor cells and TAMs may exert distinct characteristics in relation to CD8^+^ T cells. Patients with CD8^L^ + S15^H^ might be potentially applicable population if the immunosuppression was relieved in advance. These results may provide several exciting directions for future research or application of Siglec-15.

### Supplementary Information

**Additional file 1:** Supplementary Materials and Methods, Supplementary Figure S1-5, and Supplementary Table S1-4.

## Data Availability

Data used during this study are available from the corresponding author on reasonable request.

## Abbreviations

ICIs: Immune checkpoint inhibitors
PD-1: Programmed death-1
PD-L1: Programmed death-ligand 1
NSCLC: Non-small cell lung cancer
LUAD: Lung adenocarcinoma
TIME: Tumor immune microenvironment
Siglecs: Sialic acid-binding immunoglobulin-like lectins
TIICs: Tumor infiltrating immune cells
TAMs: Tumor-associated macrophages
Teffs: Effector T cells
Tregs: T regulatory cells
CTL: Cytotoxic T lymphocyte
TMUCIH: Tianjin Medical University Cancer Institute and Hospital
TMA: Tissue microarray
*mfIHC*: Multiplex fluorescence‐based immunohistochemistry
GSVA: Gene set variation analysis
TC: Tumor compartment
MC: Macrophage compartment
SC: Stroma compartment
OS: Overall survival
DFS: Disease free survival
TMIT: Tumor microenvironment immune type
APA: Acinar predominant
IMA: Invasive mucinous
LPA: Lepidic predominant
MPA: Micropapillary predominant
MIA: Minimally invasive
PPA: Papillary predominant
SPA: Solid predominant
