# Supplementary material for: Non-spatial and spatial heterogeneity revealed a suppressive immune feature of Siglec-15 in lung adenocarcinomas
Source: J Transl Med. 2023 Sep 6;21:599. doi: 10.1186/s12967-023-04489-6 (PMC10483852; doi:10.1186/s12967-023-04489-6)
Supplement: Supplementary file 1 — Additional file 1: Supplementary Materials and Methods, Supplementary Figure S1-5, and Supplementary Table S1-4. [file 12967_2023_4489_MOESM1_ESM.docx]

**Non-spatial and spatial heterogeneity revealed a suppressive immune feature of Siglec-15 in lung adenocarcinomas**

Baihui Li ^1, 2, 3, 4, 5#^, Yan Guo ^1, 2, 3, 4#^, Yeran Yi ^1, 2, 3, 4^, Ziqi Huang ^1, 2, 3, 4^, Yulin Ren ^1, 2, 3, 4^, Hao Wang ^1, 2, 3, 4^, Lili Yang ^1, 2, 3, 4*^

^1^ Department of Immunology, Tianjin Medical University Cancer Institute and Hospital, Tianjin, China.

^2^ Tianjin Medical University Cancer Institute & Hospital, National Clinical Research Center for Cancer, Tianjin, China.

^3^ Key Laboratory of Cancer Immunology and Biotherapy, Tianjin, China.

^4^ Tianjin's Clinical Research Center for Cancer, Tianjin, China.

^5^ Department of Esophageal Cancer, Tianjin Medical University Cancer Institute and Hospital, Tianjin, China.

^#^ These authors contributed equally to this work and share first authorship

****Corresponding author:***

**Lili Yang Ph.D**

Huanhuxi Road, Tiyuanbei, Hexi District, Tianjin, P. R. China, 300060

Phone: 086-022-23340123; Fax: 086-022-23537796

yanglili@tjmuch.com

**Additional files:**

Number of tables: 4;

Number of figures: 5;

**Materials and Methods**

**Patient inclusion criteria and tissue microarray (TMA) construction**

Inclusion criteria: (1) age range: 18-80 years old; (2) underwent R0 resection; (3) clear pathological diagnosis; (4) complete case records; (5) without other important related diseases (such as other malignant tumors, severe heart and central nervous system diseases, etc.); (6) without other treatment before surgery, including radiotherapy, chemotherapy, hormone therapy, targeted therapy, and other anti-tumor drug therapy; (7) clear consciousness, able to understand and answer questions clearly; (8) has signed the informed consent. The pathological diagnosis and stage of LUAD were classified according to the 8th edition of the TNM stage of the International Association for the Study of Lung Cancer. Patient informed consent was obtained and the study was approved by the Ethics Committee.

The wax blocks of tumor tissue of lung cancer patients were selected for HE staining and then histologically analyzed by a pathologist. According to the results of HE staining, representative areas in the paraffin specimens were labeled, which included typical tumor areas and stroma areas, excluding inflammation, necrotic areas, normal areas, and invasive margin. The location of the extracted tumor samples was marked on the HE slides and corresponding paraffin blocks, respectively. And then it was fixed in the TAM master blocks by puncturing with a 2.0 mm inner diameter stainless steel and the TMA blocks were embedded using a special tissue microarray embedding machine. Thus, each tumor core on the TMA block came from different patients.

**Multiplex fluorescence‐based immunohistochemistry (*mfIHC*)**

Multiplexed immunofluorescence (mIF) staining was performed based on the manufacturer’s protocol (PerkinElmer, Opal® Kit) to visualize 8 specific cell markers (Table S2), including CD163, CD68, Pan-CK, PD-L1, and Siglec-15 in PANEL-1 (n = 213); CD4, CD8, FoxP3, CD68, Pan-CK, PD-L1, and Siglec-15 in PANEL-2 (n = 189). Different primary antibodies were sequentially applied, followed by secondary antibody (poly-HRP-Ms/Rb) incubation and corresponding fluorophore using tyramide signal amplification (TSA). The slides were microwave heat-treated after each TSA operation. At last, Nuclei were stained with DAPI and the slides were covered by anti-fluorescence-quenching sealing agent and coverslips after all the human antigens had been labeled.

**Additional tables:**

| **Table S1** Patient characteristics in TMUCIH and Xinchao cohorts. | | | | |  |
| --- | --- | --- | --- | --- | --- |
| Characteristic, n (%) | TMU cohort | | Validation cohort | |  |
|  |  |  |  |  |  |
|  | LUAD | | Xinchao 04 | Xinchao 07 |  |
|  | (n=213) | | (n=83) | (n=68) |  |
| Age (year) | |  |  |  |  |
| <60 | 110(51.6) | | 27(32.5) | 31(45.6) |  |
| ≥60 | 103(48.4) | | 56(67.5) | 37(54.4) |  |
| Gender |  | |  |  |  |
| male | 92(43.2) | | 49(59.0) | 39(57.4) |  |
| female | 121(56.8) | | 34(41.0) | 29(42.6) |  |
| Smoke |  | |  |  |  |
| no | 137(64.3) | | ─ | ─ |  |
| yes | 76(35.7) | | ─ | ─ |  |
| Clinical stage |  | |  |  |  |
| Ⅰ-Ⅱ | 140(65.7) | | 53(63.9) | 44(65.7) |  |
| Ⅲ | 73(34.3) | | 30(36.1) | 23(34.3) |  |
| T classification |  | |  |  |  |
| T1 | 111(52.1) | | 20(27.4) | 15(22.7) |  |
| T2-T4 | 102(47.9) | | 53(72.6) | 51(77.3) |  |
| N classification |  | |  |  |  |
| N0 | 135(63.4) | | 50(60.2) | 30(54.5) |  |
| N1-N2 | 78(36.6) | | 33(39.8) | 25(45.5) |  |
| Pathological type |  | |  |  |  |
| APA | 74(34.7%) | | - | - |  |
| IMA | 3(1.4%) | | - | - |  |
| LPA | 27(13%) | | - | - |  |
| MPA | 48(22.5%) | | - | - |  |
| MIA | 7(3.2%) | | - | - |  |
| PPA | 11(5.1%) | | - | - |  |
| SPA | 27(12.6%) | | - | - |  |
| Missing | 16(7.5%) | | - | - |  |
|  |  | |  |  |  |
|  |  | |  |  |  |
|  |  | |  |  |  |
|  |  | |  |  |  |
|  |  | |  |  |  |

**Table S2** Opal multiplex staining protocol.

|  | Antigen | Primary antibody Provider | Catalogue number | TSA fluorophore |
| --- | --- | --- | --- | --- |
| PANEL-1 |  |  |  |  |
|  | CD163 | Abcam | ab182422 | Opal 690 |
|  | Pan-CK | Abcam | ab27988 | Opal 650 |
|  | CD68 | Invitrogen | 14-0688-82 | Opal 620 |
|  | Siglec-15 | Affinity Bioscience | BF8008 | Opal 520 |
|  | PD-L1 | Invitrogen | MA5-27896 | Opal 570 |
| PANEL-2 |  |  |  |  |
|  | CD4 | Abcam | ab133616 | Opal 480 |
|  | CD8 | Invitrogen | MA5-14548 | Opal 620 |
|  | Pan-CK | Abcam | ab27988 | Opal 690 |
|  | FoxP3 | Invitrogen | 14-4776 | Opal 650 |
|  | Siglec-15 | Affinity Bioscience | BF8008 | Opal 520 |
|  | CD68 | Invitrogen | 14-0688-82 | Opal 540 |

**Table S3** Association between Siglec-15 expression in TC and clinical parameters in LUAD in TMUCIH cohort.

| **Characteristics** | **No.of cases(%)** | **S15 in TC** | | **Positive Rate** | ***P*-value** |
| --- | --- | --- | --- | --- | --- |
|  |  | **Low** | **High** |  |  |
| Age (year) |  |  |  |  |  |
| <60 | 110(51.6) | 92 | 18 | 16.36% | 0.869 |
| ≥60 | 103(48.4) | 87 | 16 | 15.53% |  |
| Gender |  |  |  |  |  |
| male | 92(43.2) | 75 | 17 | 18.47% | 0.382 |
| female | 121(56.8) | 104 | 17 | 14.04% |  |
| Smoke |  |  |  |  |  |
| no | 137(64.3) | 116 | 21 | 15.33% | 0.734 |
| yes | 76(35.7) | 63 | 13 | 17.11% |  |
| Clinical stage |  |  |  |  |  |
| Ⅰ-Ⅱ | 140(65.7) | 121 | 19 | 13.57% | 0.187 |
| Ⅲ | 73(34.3) | 58 | 15 | 20.55% |  |
| T classification |  |  |  |  |  |
| T1 | 111(52.1) | 94 | 17 | 15.31% | 0.788 |
| T2-T4 | 102(47.9) | 85 | 17 | 16.66% |  |
| N classification |  |  |  |  |  |
| N0 | 135(63.4) | 116 | 19 | 14.07% | 0.322 |
| N1-N2 | 78(36.6) | 63 | 15 | 19.23% |  |

**Table S4** Association between Siglec-15 expression in MC and clinical parameters in LUAD in TMUCIH cohort.

| **Characteristics** | **No.of cases(%)** | **S15 in MC** | | **Positive Rate** | ***P*-value** |
| --- | --- | --- | --- | --- | --- |
|  |  | **Low** | **High** |  |  |
| Age (year) |  |  |  |  |  |
| <60 | 110(51.6) | 82 | 28 | 25.45% | 0.656 |
| ≥60 | 103(48.4) | 74 | 29 | 28.15% |  |
| Gender |  |  |  |  |  |
| male | 92(43.2) | 65 | 27 | 29.34% | 0.457 |
| female | 121(56.8) | 91 | 30 | 24.79% |  |
| Smoke |  |  |  |  |  |
| no | 137(64.3) | 100 | 37 | 27.00% | 0.913 |
| yes | 76(35.7) | 56 | 20 | 26.31% |  |
| Clinical stage |  |  |  |  |  |
| Ⅰ-Ⅱ | 140(65.7) | 104 | 36 | 25.71% | 0.633 |
| Ⅲ | 73(34.3) | 52 | 21 | 28.76% |  |
| T classification |  |  |  |  |  |
| T1 | 111(52.1) | 82 | 29 | 26.12% | 0.827 |
| T2-T4 | 102(47.9) | 74 | 28 | 27.45% |  |
| N classification |  |  |  |  |  |
| N0 | 135(63.4) | 101 | 34 | 25.18% | 0.494 |
| N1-N2 | 78(36.6) | 55 | 23 | 29.48% |  |

**Figure S1 HE and IHC representative images of LUAD in TMUCIH cohort.**

**
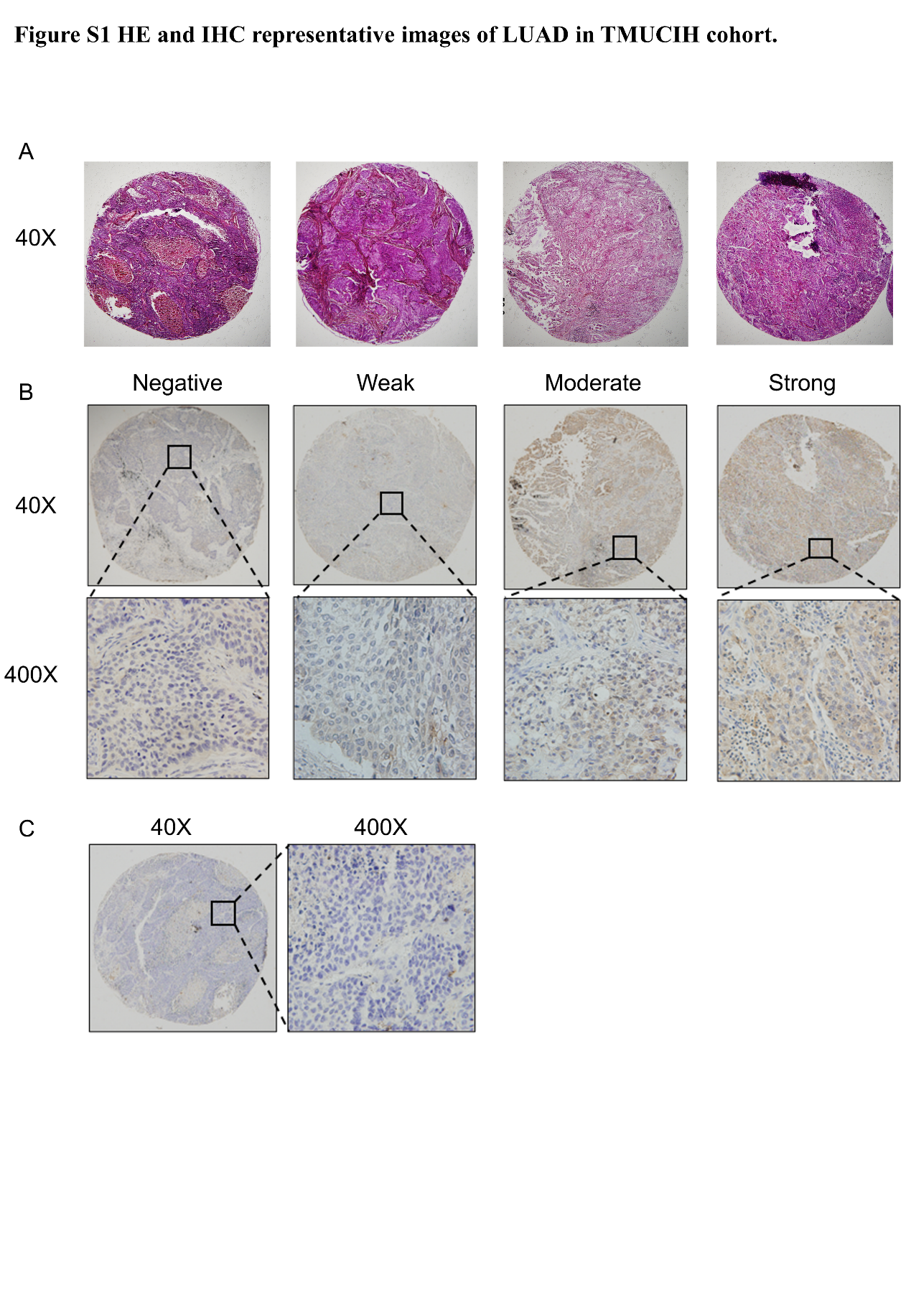
**

**Figure S2 Characteristics of M2 macrophages in LUAD in TMUCIH cohort, and the prognosis value of Siglec-15 in two validation cohorts.**

**
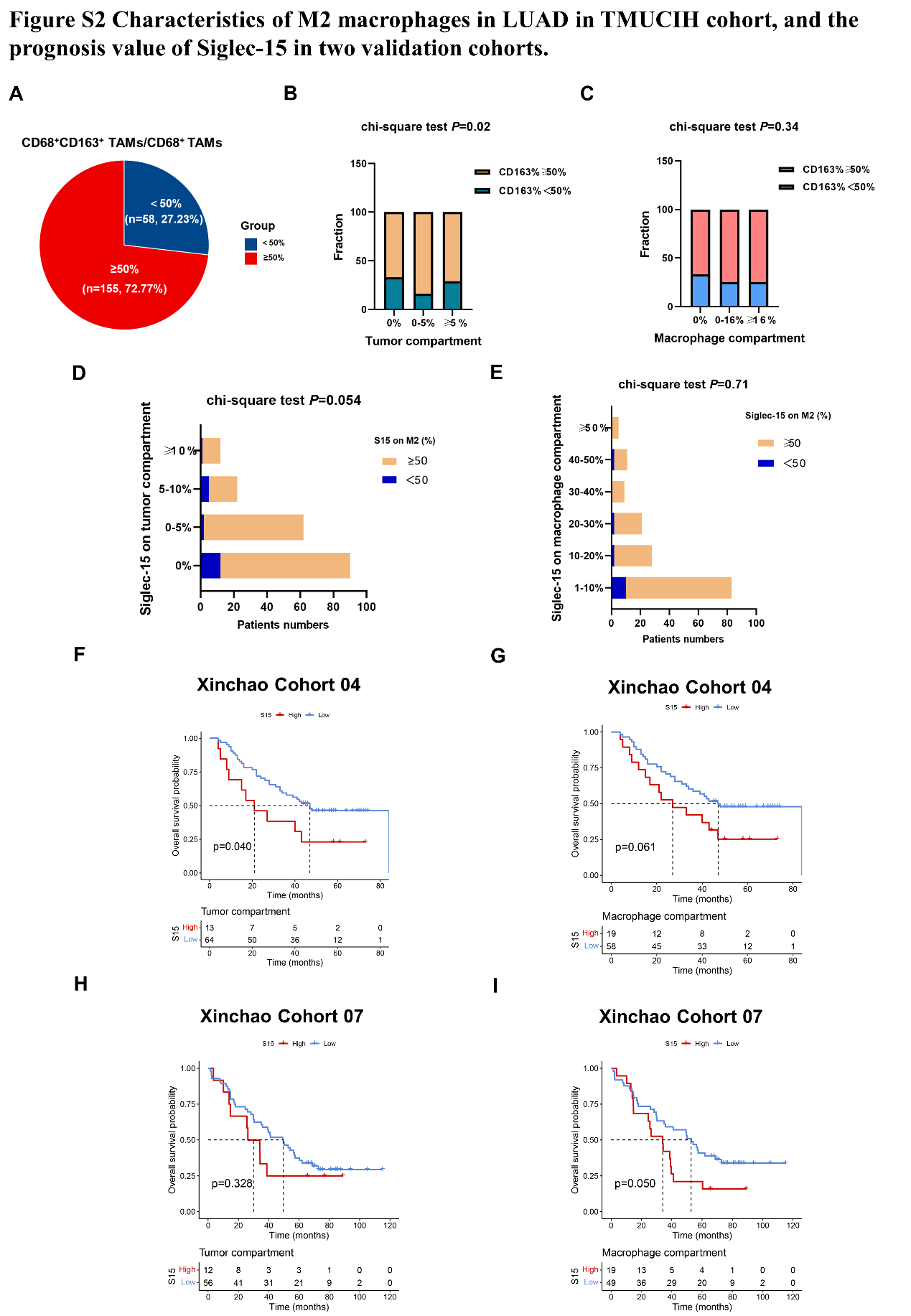
**

**Figure S3 Relationship between Siglec-15 and CD4^+^ T cells or CD4^+^Fox3^-^ Teffs in LUAD in TMUCIH cohort, and relationship between Siglec-15 and TIICs in Xinchao Cohort 04.**

**
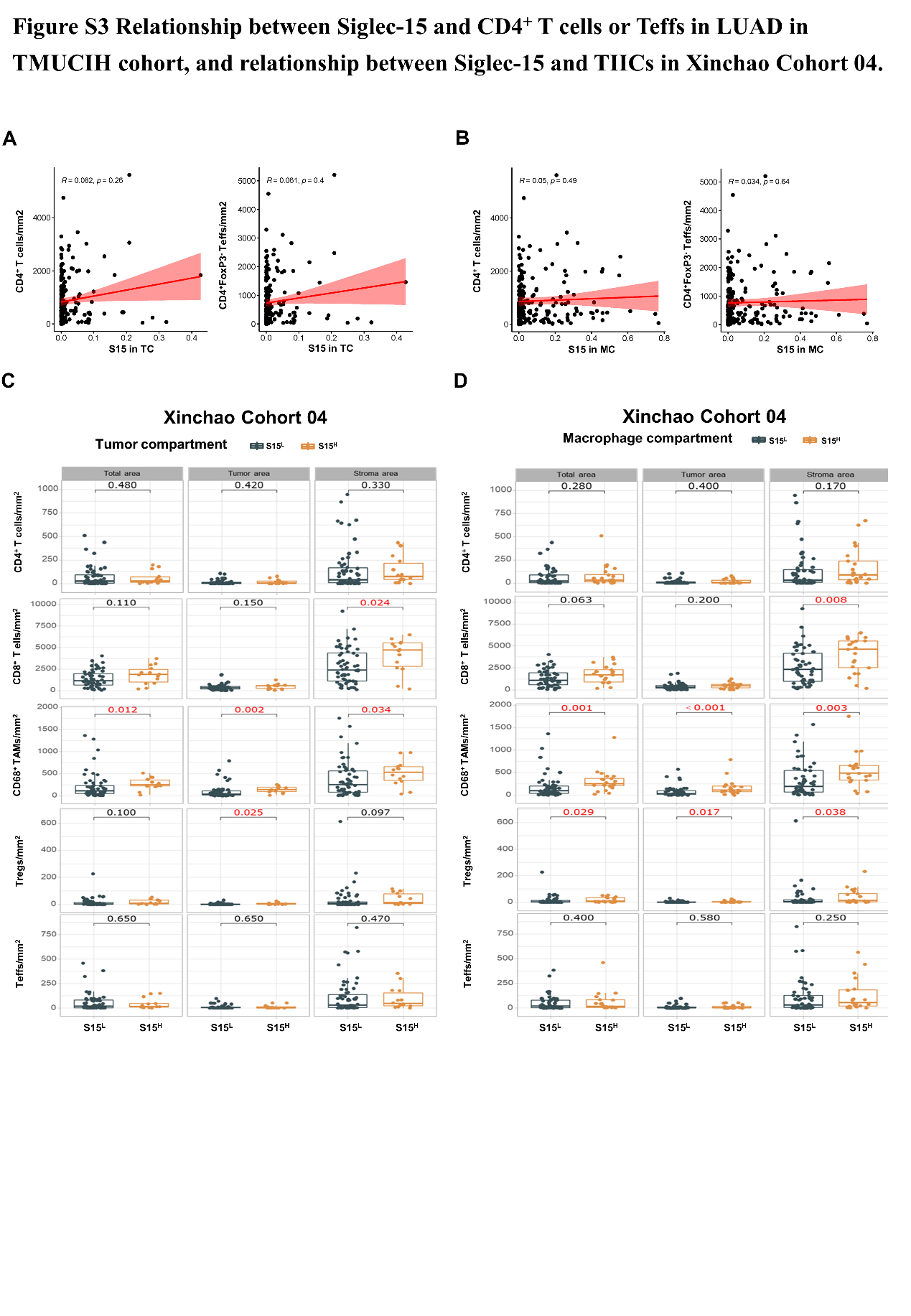
**

**Figure S4 Siglec-15^+^ tumor cells or TAMs do not affect the spatial distribution of CD8^+^ T cells, Tregs, and TAMs in PD-L1^+^ cells**

**
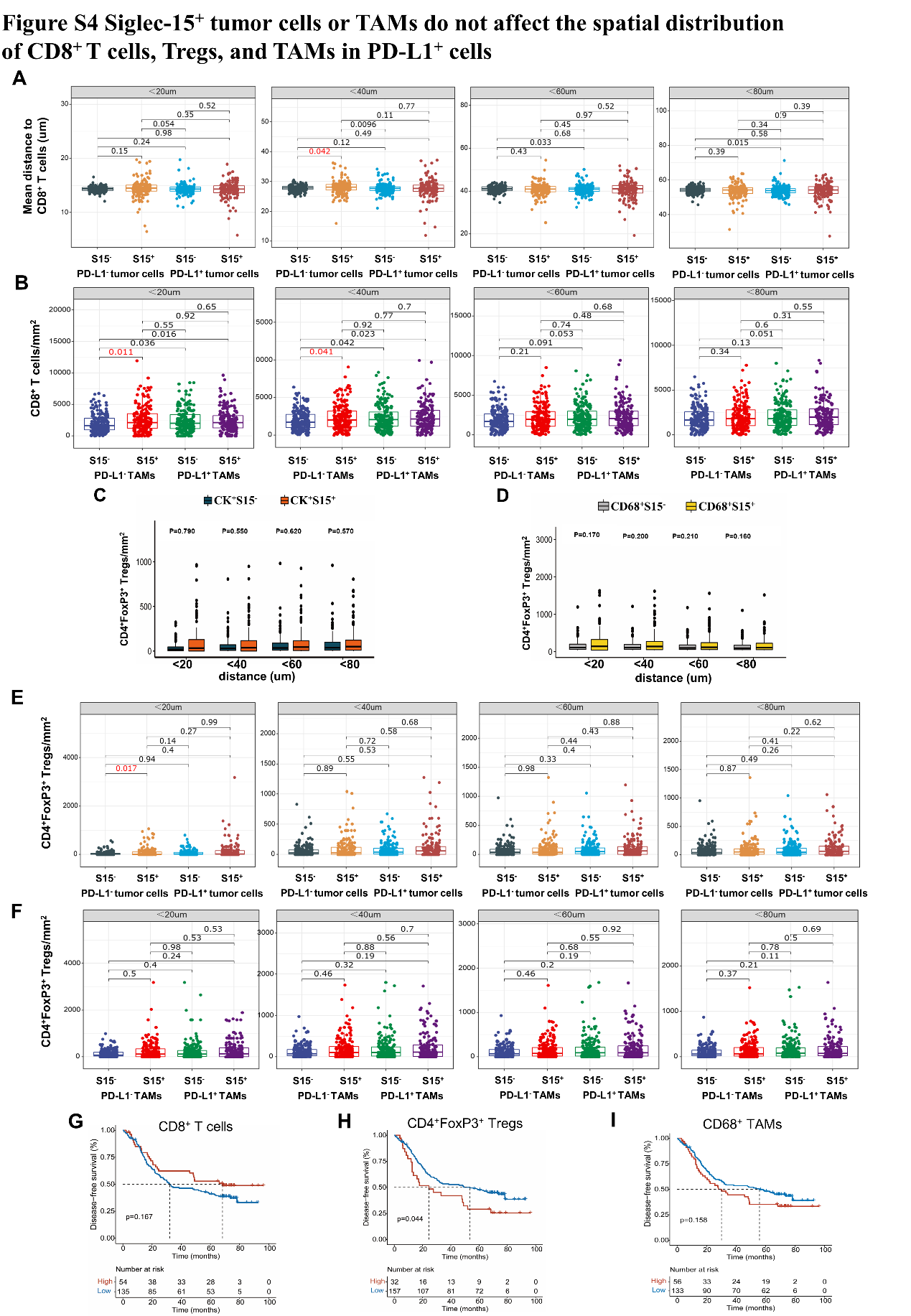
**

**Figure S5 Supplement to TMIT based on Siglec-15 and CD8^+^ T cells.**

**
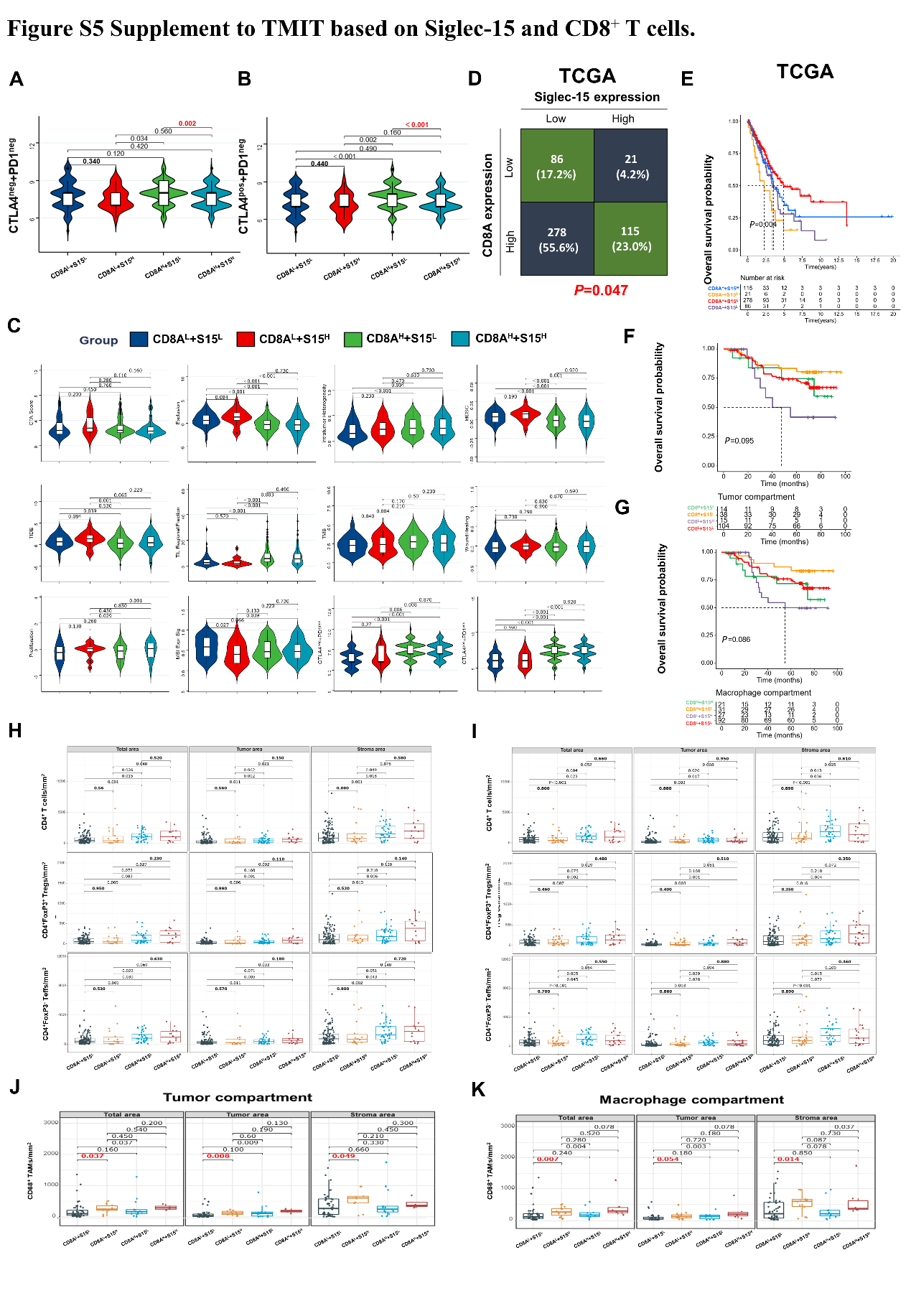
**

**Additional figures：**

**Figure S1 HE and IHC representative images of Siglec-15 in LUAD in TMUCIH cohort.**

**(A)** Representative HE staining of LUAD. **(B)** Representative pictures of immunohistochemistry staining of Siglec-15, including negative expression, weak positive, moderate positive and strong positive. **(C)** Negative control of the IHC.

**Figure S2 TAMs characteristics** **in TMUCIH cohort, and the prognosis value of Siglec-15 in two validation cohorts.**

**(A)** The percentage of M2 macrophages was more than 50% in 72% of patients in LUAD in TMUCIH cohort. **(B)** The percentage of M2 macrophages was more than 50% in groups based on the Siglec-15 expression in the TC, **(C)** and MC. **(D)** There were no difference in the proportion of Siglec-15 expression on the M2 macrophages subtype among groups based on the Siglec-15 expression in the TC, **(E)** and MC. **(F)** Siglec-15 in the TC had a prognostic value in LUAD OS in the Xinchao cohort 04. **(G)** No significant effect of Siglec-15 in the MC on LUAD OS was observed in patients in the Xinchao cohort 04. **(H)** No significant effect of Siglec-15 in the TC, or **(I)** MC on LUAD OS was observed in patients in the Xinchao cohort 07.

**Figure S3 Relationship between Siglec-15 and TIICs in LUAD in TMUCIH cohort, and Xinchao Cohort 04.**

**(A)** There was no significant correlation between Siglec-15 and CD4^+^ T cells or CD4^+^ FoxP3^-^ Teffs in the TC, **(B)** or MC. **(C)** Patients with S15^H^ in the TC had more CD8^+^ T cells than those with S15^L^ in the stroma area, more TAMs in the total, tumor, or stroma areas, and more Tregs in the tumor area. **(D)** Patients with S15^H^ in the MC had more CD8^+^ T cells than those with S15^L^ in the stroma area, more TAMs and Tregs in the total, tumor, or stroma areas.

**Figure S4 Siglec-15^+^ tumor cells or TAMs do not affect the spatial distribution of CD8^+^ T cells, Tregs, and TAMs in PD-L1^+^ cells.**

**(A)** Spatial proximity distance analysis revealed that PD-L1^-^+S15^+^ tumor cells showed slightly farther distance to CD8^+^ T cells than PD-L1^-^+S15^-^ tumor cell at a distance of less than 40 um. **(B)** In the PD-L1^-^ cells, more CD8^+^ T cells were infiltrated surrounding PD-L1^-^+S15^+^ TAMs than PD-L1^-^+S15^-^ TAMs, only in the range of less than 20, 40 um. **(C)** No difference in CD4^+^FoxP3^+^ Tregs density was observed between S15^-^ and S15^+^ tumor cells. **(E)** In the PD-L1^-^ cells, more CD4^+^FoxP3^+^ Tregs were infiltrated surrounding PD-L1^-^+S15^+^ tumor cells than PD-L1^-^+S15^-^ tumor cells, only in the range of less than 20um. **(D, F)** No difference in CD4^+^FoxP3^+^ Tregs density was observed between S15^-^ and S15^+^ TAMs. **(G)** CD8^+^ T cells had no significant effect on patient survival in the TME (*P* = 0.167). **(H)** CD4^+^FoxP3^+^ Tregs were associated with a bad prognosis in the TME (*P* = 0.044). **(I)** CD68^+^ TAMs had no prognostic significance (*P* = 0.158).

**Figure S5 Supplement to TMIT based on Siglec-15 and CD8^+^ T cells.**

**(A)** Patients with S15^H^ had a lower IPS of CTLA4^neg^+PD-1^neg^, **(B)** and CTLA4^pos^+PD-1^neg^. **(C)** No differences were found in the other signatures between the groups in TMIT. **(D)** 23% patients had CD8^H^+S15^H^, 4.2% patients had CD8^L^+S15^H^, 55.6% patients had CD8^H^+S15^L^, and 17.2% patient had CD8^L^+S15^L^. **(E)** Patients in the CD8^L^+S15^H^ group had the worst prognosis, while patients in the CD8^H^+S15^L^ group had the best prognosis. **(F)** Patients with CD8^L^+S15^H^ group had the worst OS prognosis in the TC. **(G)** Patients with CD8^L^+S15^H^ group had the worst OS prognosis in the MC. **(H)** There were no significant differences in the infiltration of CD4^+^ T cells, CD4^+^FoxP3^+^ Tregs, and CD4^+^FoxP3^-^ Teffs between groups in the TC. **(I)** There were no significant differences in the infiltration of CD4^+^ T cells, CD4^+^FoxP3^+^ Tregs, and CD4^+^FoxP3^-^ Teffs between groups in the MC. **(J)** In the case of low CD8^+^ T cell infiltration, the CD68^+^ TAMs density tended to be increased in the S15^H^ group compared with the S15^L^ group in the TC, **(K)** and MC in Xinchao 04 cohort.
